# GALNT7 Stratifies dMMR/MSI Colorectal Cancer into Distinct Molecular Subsets Associated with Prognosis and PD-L1 Expression

**DOI:** 10.1158/2767-9764.CRC-25-0270

**Published:** 2025-09-05

**Authors:** Hiroya Suzuki, Hirokazu Okayama, Shotaro Nakajima, Katsuharu Saito, Ryo Kanoda, Yuya Maruyama, Akira Matsuishi, Takuro Matsumoto, Misato Ito, Shun Chida, Wataru Sakamoto, Motonobu Saito, Zenichiro Saze, Tomoyuki Momma, Kosaku Mimura, Koji Kono

**Affiliations:** 1 Department of Gastrointestinal Tract Surgery, Fukushima Medical University School of Medicine, Fukushima, Japan.; 2 Department of Multidisciplinary Treatment of Cancer and Regional Medical Support, Fukushima Medical University School of Medicine, Fukushima, Japan.; 3 Department of Blood Transfusion and Transplantation Immunology, Fukushima Medical University School of Medicine, Fukushima, Japan.

## Abstract

Colorectal cancer with deficient mismatch repair (dMMR)/microsatellite instability (MSI) constitutes a distinct clinicopathologic and immunologic subtype, characterized by high sensitivity to immune checkpoint inhibitors. However, prognosis and therapeutic response vary considerably among dMMR/MSI colorectal cancers, underscoring the need for molecular markers to refine patient stratification. In this study, we systematically investigated cancer cell–intrinsic expression profiles of 188 glycosyltransferase genes by integrating single-cell, bulk, and cell line RNA sequencing datasets. This approach identified five glycosyltransferases, including GALNT7, expression of which differed consistently according to MSI status. The clinical and prognostic relevance of these glycosyltransferases was further analyzed across large-scale transcriptomic, proteomic, and IHC cohorts, comprising 662 dMMR/MSI and 3,483 proficient mismatch-repair (pMMR)/microsatellite-stable (MSS) colorectal cancers. A five-gene glycosyltransferase signature effectively distinguished MSI from MSS colorectal cancers across 18 datasets. Among the five genes, *GALNT7* expression was robustly associated with favorable prognosis in four independent transcriptomic and IHC cohorts of dMMR/MSI colorectal cancers while showing little or no prognostic impact in pMMR/MSS colorectal cancers. Notably, GALNT7 expression was inversely correlated with PD-L1 expression at both the mRNA and protein levels in multiple datasets exclusively within dMMR/MSI colorectal cancers, but not in pMMR/MSS CRCs. Functional assays and lectin microarray analysis using MSI colorectal cancer cell lines revealed that GALNT7 knockdown enhanced IFNγ-induced PD-L1 expression without altering cell-surface glycosylation. In conclusion, GALNT7 expression stratified dMMR/MSI colorectal cancers into distinct subsets with differential tumor cell PD-L1 expression and diverse survival outcomes, highlighting its potential as a prognostic biomarker to guide treatment strategies.

**Significance:**

We identified glycosyltransferases with altered expression depending on MMR/MSI status. Our findings indicate the existence of two molecularly defined subtypes within dMMR/MSI colorectal cancers based on GALNT7 expression, characterized by differential tumor cell PD-L1 levels and distinct survival outcomes.

## Introduction

Colorectal cancer remains the second leading cause of cancer-related deaths worldwide (1). Approximately 15% of all colorectal cancers and 5% of metastatic colorectal cancers harbor deficient mismatch repair (dMMR), resulting in microsatellite instability (MSI), whereas the remaining cases are microsatellite stable (MSS) and exhibit proficient mismatch repair (pMMR; refs. 2, 3). dMMR/MSI colorectal cancers are hypermutated and generate abundant mutation-derived neoantigens, which elicit robust immune responses, particularly through increased infiltration of CD8^+^ tumor-infiltrating lymphocytes (TIL) within the tumor microenvironment (TME; refs. 4, 5). This pronounced tumor immunogenicity, coupled with the elevated presence of TILs, is considered to contribute to the favorable prognosis observed in patients with earlier-stage dMMR/MSI colorectal cancer (2, 5–7). In contrast, metastatic dMMR/MSI colorectal cancers are more aggressive and often exhibit poorer survival outcomes compared with pMMR/MSS colorectal cancers (2, 7–9). Over the past decade, the advent of immune checkpoint inhibitors (ICI) has revolutionized the treatment landscape for dMMR/MSI colorectal cancers (3, 7). In these tumors, co-inhibitory receptors such as PD-1 and CTLA-4 on T cells, along with their ligands, including PD-L1 on tumor cells, are substantially upregulated (4, 5). This highly immunoreactive TME underpins the role of dMMR/MSI as an established biomarker for predicting responses to ICIs targeting PD-1 and/or CTLA-4 (5). In the metastatic setting, ICIs have demonstrated remarkable and durable responses with significant survival benefit in clinical trials (10–12). Nevertheless, the response to ICIs remains inconsistent, with roughly half of patients with dMMR/MSI colorectal cancer showing resistance to anti–PD-1 immunotherapy (7). These findings suggest that dMMR/MSI colorectal cancers represent a clinically heterogeneous population in terms of prognosis and therapeutic response, likely reflecting underlying molecular diversity.

Altered cell-surface glycosylation is a hallmark of malignant transformation, and some tumor-associated glycans, such as CA19-9, are widely utilized as serologic cancer biomarkers (13). Glycosylation involves the sequential actions of numerous glycosyltransferases, expression of which is tightly controlled in a cell type–specific manner, regulating diverse physiologic processes in all living cells. Altered glycan structures on cancer cells are primarily attributed to the dysregulation of glycosyltransferases, particularly at the transcriptional levels, thereby contributing to cellular phenotypes (13). Tumor-associated glycans on cancer cells not only promote cell growth, invasion, and metastasis but often induce inhibitory immune responses through interactions with glycan-binding receptors (lectins) on immune cells (13–15). For instance, Tn antigen, a tumor-associated truncated O-glycan structure, interacts with macrophage galactose-specific lectin on antigen-presenting cells, resulting in anti-inflammatory IL-10 production and effector T-cell apoptosis, thus contributing to immune evasion (14). In addition to these direct immunosuppressive effects mediated by glycans via lectin interactions, N-glycosylation of PD-L1 protein on cancer cells, likely through dysregulation of glycosyltransferases, can also contribute to the immune evasion by stabilizing PD-L1 protein structure and potentiating the PD-1/PD-L1 axis (16–18). This growing body of evidence highlights the importance of glycosylation in shaping the immunosuppressive TME. However, it remains largely unknown whether the glycosyltransferase expression profile in dMMR/MSI colorectal cancer cells differs from those in pMMR/MSS colorectal cancer cells and how such differences in glycosyltransferase activity might affect cell-surface glycan profiles, tumor biology, and clinical behavior.

Given the complex nature of the glycosylation machinery, elucidating the functional and clinical implications of glycosyltransferases within cancer cells remains challenging. We hypothesized that specific glycosyltransferases could define unique subsets of dMMR/MSI colorectal cancers associated with survival outcomes and immunophenotypes. To test this hypothesis, we integrated single-cell RNA sequencing (RNA-seq) with bulk and cell line RNA-seq to systematically investigate cancer cell–intrinsic glycosyltransferase profiles in dMMR/MSI colorectal cancers. Furthermore, we validated the identified glycosyltransferase signature across multiple transcriptomic and proteomic cohorts, followed by extensive IHC analysis, encompassing a total of more than 4,000 colorectal cancer samples. Our study identified GALNT7 as a glycosyltransferase capable of stratifying patient outcomes and modulating PD-L1 levels, specifically within dMMR/MSI colorectal cancers. This finding highlights the potential of glycosyltransferases as novel biomarkers for clinical stratification in dMMR/MSI colorectal cancer.

## Materials and Methods

### RNA-seq and microarray data analysis

All datasets used in this study are summarized in Supplementary Table S1 and depicted in Supplementary Fig. S1. Single-cell and bulk transcriptomic datasets analyzed on RNA-seq or microarray platforms are publicly available. We utilized the preprocessed values obtained from each dataset. If a gene was represented by multiple probes, only the probe with the highest mean expression was used. Clinical and genetic data, including MSI status, *BRAF* mutations, tumor mutation burden (TMB), and consensus molecular subtypes (CMS) where available, were obtained from each dataset, through the Gene Expression Omnibus (RRID: SCR_005012), cBioPortal (RRID: SCR_014555), or the Colorectal Cancer Subtyping Consortium synapse data portal (RRID: SCR_006307; https://www.synapse.org/#!Synapse:syn2623706/files/), as described previously (8, 19, 20).

For screening of glycosyltransferase genes associated with MSI status, we analyzed three RNA-seq datasets of bulk tissue [The Cancer Genome Atlas (TCGA), RRID: SCR_003193; *n* = 526; ref. 21], cell line [Cancer Cell Line Encyclopedia (CCLE), RRID: SCR_013836; *n* = 54; ref. 22], and single cell (SMC; 17,469 epithelial cells; refs. 23, 24) using the list of 188 glycosyltransferase genes from 10 Kyoto Encyclopedia of Genes and Genomes gene sets (RRID: SCR_012773) in the Molecular Signatures Database (v2023.2.Hs, RRID: SCR_016863; Supplementary Table S2; ref. 25). The selection of differentially expressed genes between MSI and MSS colorectal cancer tissues/colorectal cancer cell lines/colorectal cancer epithelial cells adhered to the following criteria: (i) expressed by at least 5% of tumor epithelial cells in the SMC dataset and (ii) absolute log_2_ fold change >0.4 in all three datasets. Single-cell RNA-seq data are often characterized by a high percentage of zero counts. To ensure the robustness of our analysis and to identify genes genuinely expressed within a specific cell type, we implemented the criterion that genes must exhibit non-zero values in at least 5% of the cells within that cell type. This 5% threshold was adopted from the methodology employed in the original comprehensive analysis of the SMC dataset (24). Based on the differentially expressed genes, we developed Glyco-MSI score, which was calculated as the average of expression values for genes that were upregulated in MSI colorectal cancers minus the average of expression values for genes that were downregulated in MSI colorectal cancers. To evaluate the performance of the Glyco-MSI score, we used a total of 18 transcriptomic cohorts and a proteomic cohort, containing 587 MSI and 2,862 MSS colorectal cancers. Immune infiltration was evaluated using the 141–immune gene signature according to ESTIMATE (RRID: SCR_026090; refs. 19, 26). For survival analysis, we utilized three datasets containing more than 300 samples with survival information, including TCGA (*n* = 566), AC-ICAM (*n* = 348), and GSE39582 (*n* = 519), and analyzed overall survival (OS), progression-free survival (PFS), and relapse-free survival (RFS) in each cohort, respectively (21, 27, 28). Patients were dichotomized into high and low groups on the basis of the median expression values for each gene or the Glyco-MSI score within each dataset.

### Patient samples

We enrolled 638 patients with primary colorectal adenocarcinoma who underwent surgical resection at Fukushima Medical University Hospital between 2002 and 2021. After excluding patients who received preoperative chemotherapy or radiotherapy, we used formalin-fixed, paraffin-embedded (FFPE) whole-tissue sections obtained from 619 patients with stage 0 to IV colorectal cancer (FMU-IHC cohort; Supplementary Table S1). We also used 20 surgically resected adenoma specimens. Tumors were classified according to the Japanese Classification of Colorectal, Appendiceal, and Anal Carcinoma (Ninth Edition). Clinical and pathologic information was obtained retrospectively from medical records. For survival analysis, 453 patients who underwent curative resection were used for RFS, which was defined as the time from the date of surgery to the date of first relapse.

### IHC

IHC was performed as previously described (29). Briefly, 4-μm-thick sections were deparaffinized and rehydrated. Endogenous peroxidases were blocked and antigens retrieved, and the slides were incubated overnight at 4°C with primary rabbit polyclonal anti-GALNT7 antibody (Atlas Antibodies Cat. #HPA064243, RRID: AB_2685223; 1:500). The following primary antibodies were also used as described previously (19, 30–32): PD-L1 (Cell Signaling Technology Cat. #13684, RRID: AB_2687655; 1:400), Tn antigen (mouse; clone MLS128; FUJIFILM/Wako; 1:500), CD4 (Agilent Cat. #M7310, RRID: AB_2728838; 1: 100), CD8 (Agilent Cat. #M7103, RRID: AB_2075537; 1:100), CD163 (Leica Biosystems Cat. #NCL-L-CD163, RRID: AB_2756375; 1:1,000), Foxp3 (Abcam Cat. #ab20034, RRID: AB_445284; 1: 200), MLH1 (Agilent Cat. #M3640, RRID: AB_3331633; 1:50), PMS2 (Agilent Cat. #M3647, RRID: AB_3331634; 1:50), MSH2 (Agilent Cat. #M3639, RRID: AB_3331635; 1:50), and MSH6 (Agilent Cat. #M3646, RRID: AB_3331636; 1:400). The sections were incubated with horseradish peroxidase–conjugated antirabbit or antimouse secondary antibodies (Agilent Cat. #K4001, RRID: AB_2827819 or Agilent Cat. #K4003, RRID: AB_2630375). Peroxidase was visualized, and nuclei were counterstained.

### Assessment of staining

Digital images were acquired using a digital slide scanner (NanoZoomer-SQ, RRID: SCR_023763). The scanned images were evaluated independently by two observers (H. Suzuki and H. Okayama) who were blinded to all clinical and pathologic data. GALNT7 staining in the cytoplasm of epithelial cells was evaluated for H-score. The intensity of staining was scored as 0 (negative), 1 (weak), 2 (moderate), or 3 (strong), and the percentage of positive cells was scored as 1 (0%–25%), 2 (26%–50%), 3 (51%–75%), or 4 (76%–100%). The intensity and positivity scores were then multiplied to obtain the GALNT7 H-score (minimum 0; maximum 12). IHC assessment for tumor cell PD-L1, Tn antigen H-score, CD8^+^ TILs, CD4^+^ TILs, Foxp3^+^ TILs, and CD163^+^ macrophages was performed as described previously (19, 30, 31).

### Determination of MSI/MMR status

For the FMU-IHC cohort, we conducted either or both MSI testing and IHC for mismatch-repair (MMR) proteins (MLH1, MSH2, MSH6, and PMS2). Briefly, loss of at least one MMR protein was defined as dMMR and tumors with intact MMR protein expression as pMMR as described previously (19). For transcriptomic and proteomic cohorts, MSI status was obtained from each dataset as descried above. We defined MSI-high as MSI and combined MSI-low and MSS as MSS.

### Cell culture

Human colorectal cancer cell lines were purchased from ATCC (RKO, RRID: CVCL_0504), RIKEN Cell Bank (HCT116, RRID: CVCL_0291), JCRB Cell Bank (LoVo, RRID: CVCL_0399), and Korean Cell Line Bank (SNU407, RRID: CVCL_5058). LS180 (RRID: CVCL_0397) and SW48 (RRID: CVCL_1724) cells were obtained as previously described and authenticated by short tandem repeat analysis (Promega Japan; ref. 32). The cells were passaged no more than 15 times before a new frozen aliquot of cells was used. Lipofectamine RNAiMAX (Thermo Fisher Scientific) was used to transfect cells with 10 nmol/L of siRNA oligonucleotides of GALNT7 or scramble control (Ambion Silencer Select; s28677, s28678, and negative control #1; Thermo Fisher Scientific). After 48 hours, the transfected cells were used for further experiments. Cell proliferation assay and colony formation assay were performed as previously described (31). For IFNγ stimulation, cells were treated with 10 ng/mL of IFNγ (285-IF; Recombinant Human IFNγ Protein; R&D Systems) for 48 hours, except for LoVo cells, which was treated with 0.5 ng/mL of IFNγ.

### Western blot analysis

Total cell proteins were extracted using Pierce RIPA Buffer (Thermo Fisher Scientific) supplemented with Halt Protease Inhibitor Cocktail (Thermo Fisher Scientific) and Phosphatase Inhibitor Cocktail Solution II (FUJIFILM/Wako). The lysates were boiled in SDS-PAGE Sample Loading Buffer (G-Biosciences). Ten micrograms of proteins was separated on 4% to 20% Tris-Glycine Mini Gel (Thermo Fisher Scientific) and transferred to polyvinylidene difluoride membranes. The membranes were blocked with 5% milk and incubated overnight at 4°C with the following primary antibodies: GALNT7 (Atlas Antibodies Cat. #HPA064243, RRID: AB_2685223; 1:1,000), PD-L1 (Cell Signaling Technology Cat. #13684, RRID: AB_2687655; 1:1,000), and β-actin (Santa Cruz Biotechnology Cat. #sc-69879, RRID: AB_1119529; 1:5,000). The membranes were then incubated with horseradish peroxidase–conjugated anti–mouse or anti–rabbit IgG antibodies (Cell Signaling Technology) for 1 hour at room temperature. Immunoreactive proteins were detected using ImageQuant LAS 4000 mini (FUJIFILM) with ECL prime Western Blotting Detection Reagent (Cytiva).

### Lectin microarray

Lectin microarray was conducted as described elsewhere (33). Briefly, membrane fractions of cells were obtained using the CelLytic MEM Protein Extraction Kit (Merck). Proteins were extracted with a Zeba Desalting Spin Column (Thermo Fisher Scientific), labeled with Cy3 NHS Ester Mono-reactive Dye Pack (Cytiva), and transferred onto a lectin microarray glass slide (LecChip 45-uni; E3100; Precision System Science). Lectin binding signals were measured using a GlycoStation Reader 2200 (emukk), and the data were analyzed using GlycoStation ToolsPro Ver.3.0 (emukk).

### Flow cytometry

Suspensions of colorectal cancer cell lines were incubated with anti–Tn antigen (mouse; clone MLS128; FUJIFILM/Wako; 1:100), followed by staining with goat anti–mouse IgG H&L (Abcam Cat. #ab150113, RRID: AB_2576208; 1:2,000). Data were acquired on FACSCanto II (BD Biosciences) and analyzed using FlowJo software (version 10.8.1, RRID: SCR_008520).

### Statistical analysis

The Fisher exact test, *χ*^2^ test, unpaired *t* test with Welch correction, or Mann–Whitney U test was used to determine differences between two variables, where appropriate. For comparisons across multiple groups, one-way ANOVA or the Kruskal–Wallis test with the Dunn *post hoc* test was performed. Correlations were assessed using the Pearson coefficient. Cumulative survival was estimated using the Kaplan–Meier method, and differences between two groups were analyzed using the log-rank test. Cox proportional hazard regression was used to compute univariable and multivariable HRs and 95% confidence intervals (CI). Statistical analyses were performed using GraphPad Prism 9 (RRID: SCR_002798). A value of *P* < 0.05 was considered to be significant.

### Ethics approval

This study was conducted in compliance with the Declaration of Helsinki. The study was approved by the Institutional Review Board of Fukushima Medical University (numbers 2289 and REC2024-041), and samples were obtained with the patients’ informed consent.

### Data availability

The data analyzed in this study were obtained from Gene Expression Omnibus (http://www.ncbi.nlm.nih.gov/geo) or cBioPortal (http://www.cbioportal.org/; ref. 34). The datasets included TCGA, CCLE, SMC (GSE132465), AC-ICAM, GSE39582, GSE26682, GSE75315, GSE41258, GSE13294, GSE24551, GSE33113, GSE42284, GSE143985, GSE4554, GSE13067, GSE39084, GSE35896, GSE18088, FOCUS-FFPE (GSE156915), CPTAC-RNA, and CPTAC-protein. All other data are available in the main article, supplemental files, or upon request from the corresponding author.

## Results

### Identification of glycosyltransferase genes associated with MSI status based on bulk, cell line, and single-cell RNA-seq analyses

**Figure 1:**
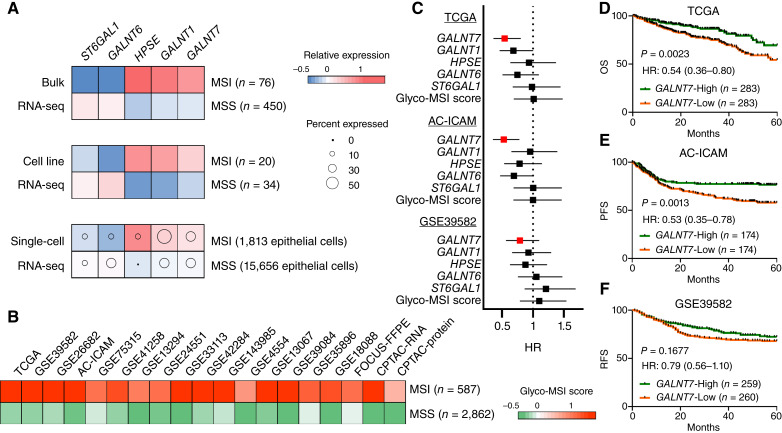
Integration of bulk, cell line, and single-cell RNA-seq for 188 glycosyltransferase genes identified five genes that were differentially expressed between MSI and MSS colorectal cancers. **A,** Heatmap depicting the upregulation of *GALNT7*, *GALNT1*, and *HPSE* and the downregulation of *GALNT6* and *ST6GAL1* in MSI colorectal cancer bulk tissues (TCGA cohort), cell lines (CCLE cohort), and single epithelial cancer cells (SMC cohort), compared with those of MSS. Dot size corresponds to the percentage of cells expressing the gene. **B,** Heatmap depicting the performance of Glyco-MSI score based on the expression of five genes to distinguish MSI from MSS colorectal cancers using 19 transcriptomic and proteomic cohorts of colorectal cancer. **C,** Forest plot demonstrating Cox HRs and 95% CIs of each five gene and the Glyco-MSI score for OS in TCGA cohort, PFS in the AC-ICAM cohort, and RFS in the GSE39582 cohort. **D–F,** Kaplan–Meier curves according to the expression of GALNT7 in TCGA (**D**), AC-ICAM (**E**), and GSE39582 (**F**). Log-rank *P* values, Cox HRs, and 95% CIs are indicated. For survival analysis, patients were dichotomized as high or low based on the median expression of *GALNT7*.

To identify glycosyltransferase genes that are differentially expressed between MSI and MSS colorectal cancer cells, we employed a hybrid screening approach integrating bulk, cell line, and single-cell RNA-seq datasets. This included TCGA cohort (76 MSI and 450 MSS colorectal cancer tissues), the CCLE cohort (20 MSI and 34 MSS colorectal cancer cell lines), and the SMC cohort (1,813 single-tumor epithelial cells obtained from 4 MSI colorectal cancer tissues and 15,636 single-tumor epithelial cells from 19 MSS colorectal cancer tissues; Supplementary Fig. S1; Supplementary Table S1), and we analyzed the expression of 188 glycosyltransferase genes included in 10 Kyoto Encyclopedia of Genes and Genomes gene sets (Supplementary Table S2). As shown in Fig. 1A and Supplementary Table S3, this analysis identified five glycosyltransferase genes, namely, *ST6GAL1*, *GALNT6*, *HPSE*, *GALNT1*, and *GALNT7*, expression of which was consistently upregulated or downregulated in MSI colorectal cancer tissues, cell lines, and single epithelial cells compared with MSS colorectal cancers. To further assess the cell type–specificity, we examined their expression not only in single epithelial cells but also in single stromal and immune cells from both tumor and normal mucosal tissues using the SMC dataset (Supplementary Fig. S2). The expression of each gene was predominantly detected in cancer epithelial cells compared with normal epithelial cells. Additionally, we observed that these genes exhibited epithelial cell–specific expression, except for *GALNT1*, which was expressed in both epithelial and stromal cells.

### Glyco-MSI score developed using five glycosyltransferase genes effectively discriminates MSI colorectal cancers from MSS colorectal cancers across 18 bulk transcriptomic and proteomic datasets

Based on the five genes, we developed an expression-based signature, designated as the Glyco-MSI score, and applied it to multiple bulk cohorts. These included 18 transcriptomic datasets analyzed using RNA-seq or microarrays and a mass spectrometry–based proteomic dataset, collectively comprising 587 MSI and 2,862 MSS samples (Supplementary Fig. S1; Supplementary Table S1). Strikingly, the Glyco-MSI score was significantly elevated in MSI colorectal cancers compared with MSS colorectal cancers across all 19 datasets, demonstrating strong discriminative performance with AUC values ranging from 0.80 to 0.97 in each cohort (Fig. 1B; Supplementary Figs. S3 and S4). These findings provide robust validation of the differential expression patterns of the five glycosyltransferases, indicating that the Glyco-MSI score can serve as a highly effective classifier for distinguishing MSI colorectal cancers within bulk colorectal cancer samples.

### GALNT7 expression is associated with survival outcomes across multiple colorectal cancer cohorts

We next sought to evaluate the prognostic significance of each of the five individual genes and the Glyco-MSI score across three large cohorts with survival data: TCGA (*n* = 566), AC-ICAM (*n* = 348), and GSE39582 (*n* = 519). In each cohort, patients were dichotomized into two groups based on the median expression of each gene or the Glyco-MSI score, and Cox hazard models were used to calculate HRs for OS, PFS, or RFS (Fig. 1C). Among the five genes, high *GALNT7* expression consistently demonstrated a trend toward favorable prognosis: TCGA (HR for OS = 0.54; 95% CI, 0.36–0.80), AC-ICAM (HR for PFS = 0.53; 95% CI, 0.35–0.78), and GSE39582 (HR for RFS = 0.79; 95% CI, 0.56–1.10). In contrast, the other four genes and the Glyco-MSI score exhibited inconsistent or nonsignificant survival associations. Consistently, Kaplan–Meier curves showed that higher *GALNT7* expression was significantly associated with better survival in both TCGA (log-rank *P* = 0.0023, Fig. 1D) and AC-ICAM (*P* = 0.0013, Fig. 1E) cohorts. A similar prognostic trend was observed in GSE39582, although it did not reach statistical significance (*P* = 0.1677, Fig. 1F). Multivariable analyses demonstrated that high *GALNT7* expression was significantly associated with better survival in TCGA and AC-ICAM cohorts but not in the GSE39582 cohort (Supplementary Table S4). We also examined the relationship between *GALNT7* expression and clinicopathologic characteristics (Supplementary Fig. S5A–S5E). Overall, *GALNT7* expression was not consistently associated with age at diagnosis, sex, tumor location, or disease stage, whereas its expression was significantly higher in mucinous adenocarcinomas compared with nonmucinous adenocarcinomas.

### GALNT7 expression has strong prognostic value in MSI colorectal cancers

**Figure 2:**
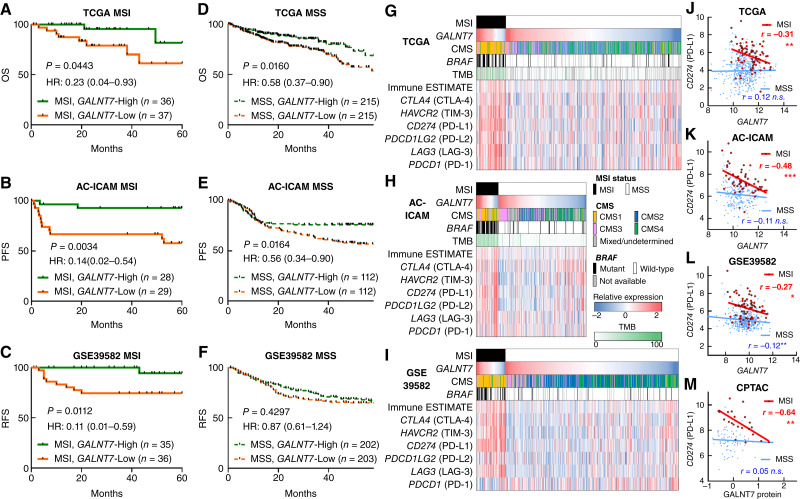
Prognostic impact of *GALNT7* and the association between *GALNT7* and PD-L1 (*CD274*) in colorectal cancer, stratified by MSI status. **A–F,** Kaplan–Meier curves for OS in TCGA cohort (**A** and **D**), PFS in the AC-ICAM cohort (**B** and **E**), and RFS in the GSE39582 cohort (**C** and **F**), analyzed separately for MSI (**A–C**) and MSS (**D–F**) colorectal cancers according to the expression of *GALNT7*. Patients were dichotomized as high or low based on the median expression of *GALNT7* in each analysis. Log-rank *P* values, Cox HRs, and 95% CIs are indicated. **G–I,** Heatmaps depicting MSI status, *GALNT7* expression, CMS, *BRAF* mutations, TMB, immune infiltration estimated using the ESTIMATE, and the expression of immune checkpoint genes in TCGA (**G**), AC-ICAM (**H**), and GSE39582 (**I**). **J–L,** Correlations between *CD274* and *GALNT7* mRNA expression according to MSI status in TCGA (**J**), AC-ICAM (**K**), and GSE39582 (**L**). **M,** Correlations between *CD274* and GALNT7 protein expression according to MSI status in CPTAC. Pearson correlation coefficients are indicated. ***, *P* < 0.001; **, *P* < 0.01; *, *P* < 0.05; n.s. *P* > 0.05.

Given that *GALNT7* expression was associated with survival outcomes and differentially expressed between MSI and MSS colorectal cancers, we hypothesized that its prognostic significance may vary depending on MSI status. Notably, despite the relatively smaller sample size of MSI colorectal cancers, *GALNT7* expression was significantly associated with survival outcomes across all MSI cohorts, including TCGA MSI (*n* = 73; log-rank *P* = 0.0443; Cox HR = 0.23; 95% CI, 0.04–0.93; Fig. 2A), AC-ICAM MSI (*n* = 57; *P* = 0.0034; HR = 0.14; 95% CI, 0.02–0.54; Fig. 2B), and GSE39582 MSI (*n* = 71; *P* = 0.0112; HR = 0.11; 95% CI, 0.01–0.59; Fig. 2C). In MSS colorectal cancers, *GALNT7* expression was associated with survival in TCGA MSS (*n* = 430; *P* = 0.0160; HR = 0.58; 95% CI, 0.37–0.90; Fig. 2D) and AC-ICAM MSS (*n* = 224; *P* = 0.0164; HR = 0.56; 95% CI, 0.34–0.90; Fig. 2E) but not in GSE39582 MSS (*n* = 405; *P* = 0.4297; HR = 0.87; 95% CI, 0.61–1.24; Fig. 2F). Importantly, the strength of this prognostic association in MSI colorectal cancers is evident from the markedly lower HR values across all three cohorts, highlighting a robust correlation between higher *GALNT7* expression and better survival. Moreover, multivariable analysis of MSI colorectal cancers demonstrated that *GALNT7* had significant prognostic value in the AC-ICAM MSI (HR = 0.20; 95% CI, 0.03–0.84; *P* = 0.047) and GSE39582 MSI (HR = 0.09; 95% CI, 0.01–0.52; *P* = 0.027) cohorts, independent of other clinical covariates, whereas it showed marginal significance in TCGA MSI cohort (HR = 0.24; 95% CI, 0.04–1.01; *P* = 0.079; Supplementary Table S4). By contrast, *GALNT7* was not associated with survival outcomes in MSS colorectal cancers in multivariable models. These findings suggest that the prognostic impact of *GALNT7* expression is primarily observed in MSI colorectal cancers, whereas its significance in MSS colorectal cancers is limited.

### GALNT7 expression correlates with the TME and the expression of immune checkpoint genes

Given that the expression of *GALNT7* had a prognostic impact, particularly in MSI colorectal cancers, we speculated that molecular and/or immune profiles might also differ according to *GALNT7* levels within MSI colorectal cancers. To explore this possibility, we investigated the potential associations between *GALNT7* expression and various genomic, transcriptomic, and immunologic features, including CMS, *BRAF* mutations, TMB, immune infiltration (assessed using immune ESTIMATE), and immune checkpoint gene expression. These analyses were conducted separately for MSI and MSS colorectal cancers using three datasets. In MSI colorectal cancers, *GALNT7* levels were not significantly associated with CMS, *BRAF* mutation, or TMB in any of the cohorts (Fig. 2G–I; Supplementary Fig. S6A–S6C). In contrast, *GALNT7* expression generally exhibited a negative correlation with the levels of immune ESTIMATE and immune checkpoint gene expression (Fig. 2G–I; Supplementary Fig. S7). Specifically, in MSI colorectal cancers, immune ESTIMATE, *HAVCR2* (TIM-3), *CD274* (PD-L1), *LAG3* (LAG-3), and *PDCD1* (PD-1) each showed significant inverse correlations with *GALNT7* expression across the three cohorts (Fig. 2J–L; Supplementary Table S5). Notably, these negative correlations between *GALNT7* and immune checkpoint genes were predominantly observed in MSI colorectal cancers, whereas MSS colorectal cancers exhibited weaker or no correlations. Furthermore, using the CPTAC cohort, we confirmed a negative correlation between GALNT7 protein levels and *CD274* expression, specifically in MSI colorectal cancers (Fig. 2M).

### GALNT7 IHC identifies dMMR/MSI colorectal cancer subtypes associated with PD-L1, Tn antigen, and prognosis

**Figure 3:**
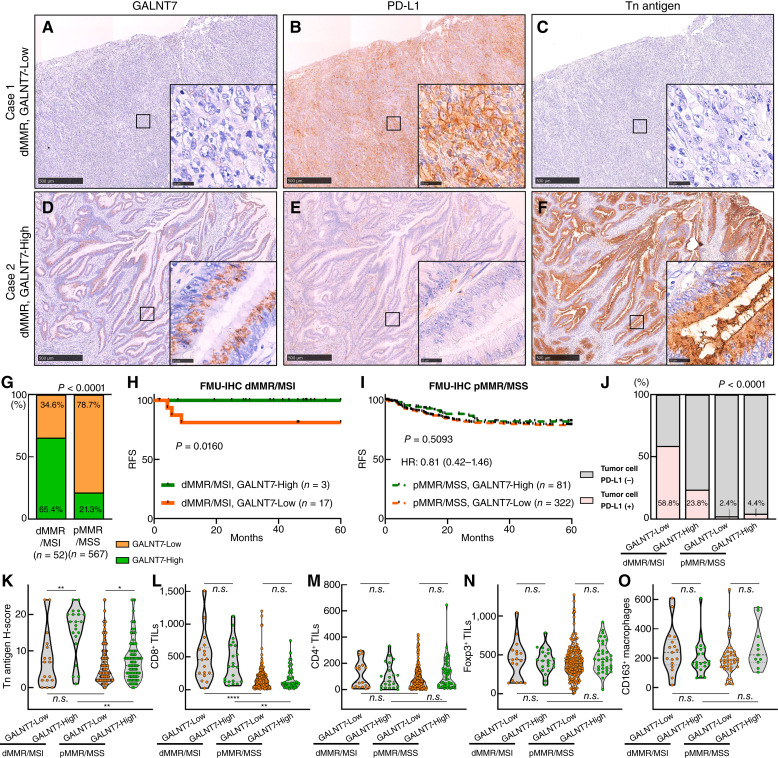
IHC for GALNT7 expression in colorectal cancer. **A–C,** Representative images of a case of dMMR colorectal cancer with GALNT7-Low (**A**), showing tumor cell PD-L1 positive (**B**) and lack of Tn antigen expression (**C**). **D–F,** Representative images of a case of dMMR colorectal cancer with GALNT7-High (**D**), showing tumor cell PD-L1 negative (**E**) and strong Tn antigen expression (**F**). **G,** Proportion of GALNT7-Low and GALNT7-High in dMMR/MSI and pMMR/MSS colorectal cancers. **H** and **I,** Kaplan–Meier curves illustrating RFS according to GALNT7 expression by IHC in dMMR/MSI (**H**) and pMMR/MSS (**I**) colorectal cancers. The log-rank *P* value, Cox HR, and 95% CI are indicated. **J,** Relative proportion of tumor cell PD-L1 positive and negative in colorectal cancer according to GALNT7 IHC and MMR/MSI status. **K,** Tn antigen H-score in colorectal cancer according to GALNT7 IHC and MMR/MSI status. **L–O,** Levels of immune infiltration of CD8^+^ lymphocytes (**L**), CD4^+^ lymphocytes (**M**), Foxp3^+^ lymphocytes (**N**), and CD163^+^ macrophages (**O**) in colorectal cancer according to GALNT7 IHC and MMR/MSI status. ****, *P* < 0.0001; **, *P* < 0.01; *, *P* < 0.05; n.s. *P* > 0.05.

Given that the prognostic impact of GALNT7 and its inverse relationship with immune checkpoint expression were observed in MSI colorectal cancers at the transcriptional level, we further sought to investigate the clinicopathologic and prognostic role of GALNT7 at the protein level using IHC (Fig. 3A–O). We analyzed FFPE specimens from 20 adenomas and 619 colorectal cancers, including 575 adjacent nontumor tissues available for evaluation. GALNT7 IHC revealed granular cytoplasmic staining in nonneoplastic epithelial cells and tumor cells at variable levels, whereas no staining was observed in stromal or immune cells (Supplementary Fig. S8A–S8H). This finding confirmed the epithelial cell–specific expression of *GALNT7* mRNA observed in single-cell analyses (Supplementary Fig. S2). The GALNT7 H-score was significantly higher in dMMR/MSI colorectal cancers compared with both nontumor mucosa and pMMR/MSS colorectal cancers (Supplementary Figs. S9 and S10A). In contrast, GALNT7 expression did not significantly change during tumor progression from adenoma to stage 0 to IV colorectal cancers (Supplementary Fig. S10B).

Based on the H-score, colorectal cancers were further classified into GALNT7-High (H-score, 8–12) or GALNT7-Low (H-score, 0–7) group. Among 52 dMMR/MSI colorectal cancers, 65.4% were classified as GALNT7-High, whereas only 21.3% of 567 pMMR/MSS colorectal cancers fell into the GALNT7-High category (Fig. 3G, *P* < 0.0001). We found no significant associations between GALNT7 IHC and clinicopathologic features, such as tumor location, tumor differentiation, tumor invasion, or lymph node metastasis (Supplementary Table S6). Although not statistically significant, mucinous histology was enriched in GALNT7-High colorectal cancers (*P* = 0.0565, Supplementary Table S6), which was consistent with the findings at transcriptional levels (Supplementary Fig. S5D). We next conducted survival analysis, and GALNT7-High colorectal cancers showed a nonsignificant trend toward favorable RFS compared with GALNT7-Low colorectal cancers (n = 453; *P* = 0.0692; HR = 0.57; 95% CI, 0.29–1.02; Supplementary Fig. S10C). Most importantly, we observed a striking difference in the prognostic impact of GALNT7 IHC between dMMR/MSI and pMMR/MSS colorectal cancers. In dMMR/MSI colorectal cancers, GALNT7-High status was significantly associated with better RFS (*n* = 50; *P* = 0.0160, Fig. 3H), whereas GALNT7 staining had no prognostic value in pMMR/MSS colorectal cancers (*n* = 403; *P* = 0.5093; HR = 0.81; 95% CI, 0.42–1.46; Fig. 3I). These findings further validated the prognostic significance of *GALNT7* mRNA expression, particularly in MSI colorectal cancers (Fig. 2A–F). Among dMMR/MSI colorectal cancers, GALNT7-Low status was associated with poor tumor differentiation but not with other clinicopathologic characteristics (Supplementary Table S7). As there is a clinical need for identifying postoperative stage II to III patients at high risk of disease recurrence, we further conducted stage-stratified RFS analyses for dMMR/MSI colorectal cancers. Supplementary Fig. S11A and S11B demonstrated that GALNT7-Low was significantly associated with shorter RFS in stage II to III colorectal cancers in both the GSE39582 MSI (*n* = 59; *P* = 0.0064) and FMU-IHC dMMR/MSI cohorts (*n* = 37; *P* = 0.0194). In the stratified analysis of stage II and stage III, GALNT7-Low expression demonstrated a significant association or a nonsignificant trend toward poorer RFS (Supplementary Fig. S11B–S11F).

We also examined the relationship between GALNT7 staining and tumor cell PD-L1 positivity, Tn antigen H-score, and infiltration of CD8^+^ TILs, CD4^+^ TILs, Foxp3^+^ TILs, and CD163^+^ macrophages, stratified by MMR/MSI status (Fig. 3A–F; Supplementary Fig. S12A–S12D). In this FFPE cohort, dMMR/MSI colorectal cancers were characterized by tumor cell PD-L1 expression, Tn antigen expression, and increased levels of CD8^+^ TILs (Supplementary Fig. S12E–S12J). Intriguingly, and consistent with the findings from transcriptomic datasets (Fig. 2J–M), dMMR/MSI colorectal cancers with GALNT7-High expression exhibited a significantly lower frequency of tumor cell PD-L1 positivity (23.8%) compared with GALNT7-Low dMMR/MSI colorectal cancers (58.8%; Fig. 3J). However, this inverse relationship was not observed in pMMR/MSS colorectal cancers. Conversely, we found a marked increase in Tn antigen H-score in GALNT7-High dMMR/MSI colorectal cancers compared with their GALNT7-Low counterparts, and this concurrent upregulation of Tn antigen and GALNT7 was also observed in pMMR/MSS colorectal cancers (Fig. 3K). Infiltration of CD8^+^ TILs, CD4^+^ TILs, Foxp3^+^ TILs, or CD163^+^ macrophages did not differ between GALNT7-High and GALNT7-Low tumors in either dMMR/MSI or pMMR/MSS colorectal cancers (Fig. 3L–O).

### GALNT7 does not regulate Tn antigen expression in MSI colorectal cancer cell lines

GALNT isoenzymes initiate O-glycosylation by adding GalNAc residues to Ser/Thr amino acids, leading to the synthesis of the Tn antigen, which can subsequently be elongated and branched to form various glycan structures (13). Given that our IHC analyses revealed an association between GALNT7-High status and increased Tn antigen expression, particularly in dMMR/MSI colorectal cancers (Fig. 3C, F, and K), we hypothesized that upregulated GALNT7 may directly influence glycan expression profiles, particularly by increasing Tn antigen levels on dMMR/MSI colorectal cancer cells, thereby contributing to cellular functions. To test this hypothesis, we conducted a series of *in vitro* assays using MSI colorectal cancer cell lines transfected with siRNAs targeting GALNT7 (Supplementary Fig. S13). However, GALNT7 knockdown had inconsistent or only a modest effect on cell proliferation and colony formation among these cell lines (Supplementary Fig. S14). Furthermore, to examine cell-surface glycan profiles, we performed lectin microarray analysis on membranous fractions obtained from cultured MSI colorectal cancer cells. Depletion of GALNT7 did not result in significant changes in any lectin binding signals in either LS180 or LoVo cells (Supplementary Fig. S15A and S15B). Notably, the signal intensities of lectins recognizing O-glycan structures, such as Tn antigen (MAH, ABA, ACA, Jacalin, and MPA), remained comparable between negative control and GALNT7-knockdown cells (Supplementary Fig. S15C and S15D). Additionally, flow cytometry analysis using a panel of MSI colorectal cancer cell lines showed that GALNT7 knockdown had no significant effect on the cell-surface Tn antigen levels (Supplementary Fig. S16). Collectively, these findings indicate that GALNT7 depletion does not significantly alter cell proliferation, cell-surface glycan profiles, or Tn antigen expression in MSI colorectal cancer cells.

### Depletion of GALNT7 leads to upregulation of PD-L1 in MSI colorectal cancer cell lines

**Figure 4:**
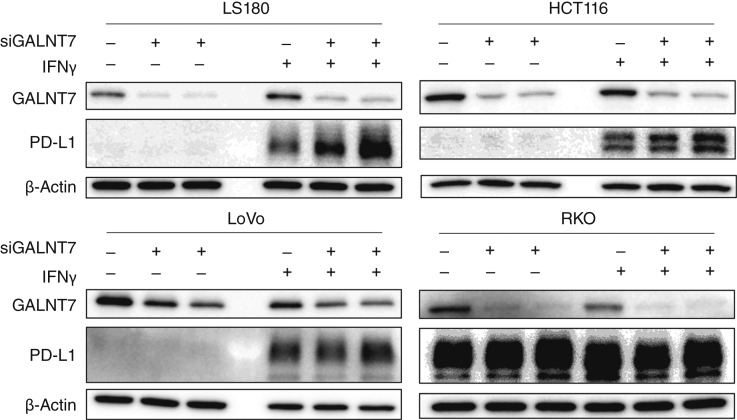
GALNT7 depletion and IFNγ-induced PD-L1 expression in MSI colorectal cancer cell lines. LS180, HCT116, LoVo, and RKO cells transfected with siRNAs targeting GALNT7 or scramble negative control in the presence or absence of IFNγ, analyzed using Western blotting.

Given that we consistently observed an inverse relationship between GALNT7 and PD-L1 levels in dMMR/MSI colorectal cancers—but not in pMMR/MSS colorectal cancers—across multiple transcriptomic, proteomic, and IHC datasets (Figs. 2J–M, and 3J), we hypothesized that GALNT7 negatively regulates PD-L1 expression in dMMR/MSI colorectal cancer cells. To test this hypothesis, we treated MSI colorectal cancer cell lines with IFNγ, which strongly induced PD-L1 expression in most cell lines, except for RKO cells, which constitutively expressed high levels of endogenous PD-L1 (Fig. 4). Western blot analysis revealed that GALNT7 knockdown resulted in increased PD-L1 levels following IFNγ stimulation in HCT116 and LS180 cells, whereas its effect was less pronounced in RKO and LoVo cells (Fig. 4).

## Discussion

To the best of our knowledge, this study represents the largest analysis to date focusing on cancer cell–intrinsic glycosyltransferase profiles in colorectal cancer in relation to MMR/MSI status, in which we analyzed a total of 662 dMMR/MSI and 3,483 pMMR/MSS colorectal cancers. Our findings align with previous studies indicating that glycosyltransferases can influence immune responses and tumor progression. However, the role of GALNT7 in dMMR/MSI colorectal cancers has remained largely unexplored. Here, we provide several novel insights. First, we conducted a gene expression analysis of 188 glycosyltransferases by integrating single-cell RNA-seq with bulk and cell line RNA-seq datasets. This approach allowed us to identify five glycosyltransferase genes that were consistently upregulated or downregulated in MSI cancer cells, MSI cell lines, and MSI tissues compared with their MSS counterparts. Second, based on these five genes, we developed the Glyco-MSI score, which effectively distinguished MSI colorectal cancers from MSS colorectal cancers with high accuracy (AUCs ranging from 0.80 to 0.97) across 19 independent cohorts. This robust finding suggests that the Glyco-MSI score could serve as a diagnostic tool for classifying MSI or MSS colorectal cancers based on bulk expression data. Third, among the five genes, *GALNT7* was significantly upregulated in MSI colorectal cancers compared with MSS CRCs. This observation was further validated by IHC, demonstrating that high GALNT7 protein expression was frequently observed in dMMR/MSI colorectal cancers, whereas pMMR/MSS colorectal cancers exhibited relatively weak GALNT7 staining, similar to that observed in nontumor mucosa. Fourth, we found that GALNT7 had a strong prognostic impact in dMMR/MSI colorectal cancers at both the mRNA and protein levels, whereas it had little or no prognostic value in pMMR/MSS colorectal cancers. Fifth, GALNT7 expression was inversely associated with PD-L1 expression at both the mRNA and protein levels, and this association was exclusively observed in dMMR/MSI colorectal cancers. The inverse relationship between GALNT7 and PD-L1 was further supported by *in vitro* experiments, in which GALNT7 depletion resulted in increased PD-L1 levels in at least two MSI colorectal cancer cell lines. Lastly, our data suggest the existence of two molecularly defined subtypes within dMMR/MSI colorectal cancers based on GALNT7 expression, characterized by differential tumor cell PD-L1 levels and distinct survival outcomes.

Patients with dMMR/MSI colorectal cancer in general have a low risk of recurrence but derive limited benefit from conventional chemotherapy, although its prognostic impact remains controversial (3, 35, 36). However, when disease recurrence and/or metastasis occurs, dMMR/MSI colorectal cancers often display even poorer survival outcomes than pMMR/MSS colorectal cancers (8, 9). Moreover, responsiveness to immunotherapy is highly variable among metastatic dMMR/MSI colorectal cancers, with approximately 50% of them displaying primary resistance to ICIs (7, 10–12). These findings suggest that dMMR/MSI colorectal cancers consist of molecularly heterogeneous populations that correlate with clinical behaviors. However, the current molecular markers, such as *BRAF* mutations, fail to stratify the prognostic and therapeutic heterogeneity within dMMR/MSI colorectal cancers (3). In this study, we investigated the intertumoral heterogeneity of dMMR/MSI colorectal cancers, and our findings indicate that GALNT7 expression serves as a robust prognostic biomarker in this subgroup. IHC for GALNT7 may effectively identify a subset of dMMR/MSI colorectal cancers characterized by high GALNT7 expression and excellent prognosis. In contrast, among dMMR/MSI colorectal cancers, the GALNT7-Low subset exhibits high levels of tumor cell PD-L1 and poor prognosis, highlighting the need for more intensive therapeutic strategies beyond current clinical practice. Additionally, because GALNT7 was negatively correlated not only with PD-L1 but also with PD-1, CTLA-4, TIM-3, and LAG-3 across multiple transcriptomic MSI cohorts, dMMR/MSI GALNT7-Low tumors are likely to express not only PD-L1 but also other immune checkpoint molecules. Our results warrant future clinical and mechanistic studies to determine whether dMMR/MSI GALNT7-Low colorectal cancers derive greater benefit from PD-1/PD-L1 blockade, alone or in combination with a CTLA-4, TIM-3, or LAG-3 inhibitor.

Our *in vitro* data demonstrated that GALNT7 depletion led to PD-L1 upregulation in HCT116 and LS180 cells following IFNγ stimulation. These findings were robustly supported by multiple cohort analyses, consistently demonstrating an inverse correlation between GALNT7 and PD-L1 expression at both the mRNA and protein levels. Mechanistically, it remains unclear how GALNT7 exerts its regulatory effects on PD-L1. Given that GALNT7 is primarily involved in O-glycosylation (O-GalNAc glycosylation), one possible explanation is that alterations in O-GalNAc modification patterns influence PD-L1 stability. Indeed, N-glycosylation of PD-L1, mediated by several glycosyltransferases, has been shown to play a crucial role in PD-L1 stability and its interaction with PD-1, thereby contributing to immune evasion (16–18). Furthermore, a recent study demonstrated that O-GlcNAc glycosylation, which modifies intracellular proteins via O-GlcNAc transferase, inhibited PD-L1 degradation in cancer cells, thereby promoting immune evasion (37). Notably, inhibition of O-GlcNAc glycosylation reduced PD-L1 levels and sensitized cancer cells to anti–PD-L1 therapy in mouse models (38). As O-GalNAc modifications of PD-L1 have not been previously characterized, further investigation is warranted to determine whether O-GalNAc glycosylation, particularly via GALNT7, contributes to a similar regulatory mechanism. Additionally, it remains to be determined whether GALNT7 influences PD-L1 expression through glycosylation-dependent or -independent pathways.

Our data highlight a potential role of GALNT7 in modulating the immunosuppressive TME. Intriguingly, in melanoma cells, GALNT7 silencing was associated with increased IL-10 secretion, enhanced CTLA-4 expression, and the modulation of immune evasion (39). Moreover, prostate cancer tissues and cell lines with decreased GALNT7 expression exhibited significant enrichment of immune-related pathways, such as immunoregulatory interaction and IFNγ response (40). A recent pan-cancer analysis across 33 cancer types further revealed that *GALNT7* expression had either a positive or negative impact on prognosis and tumor immunophenotypes, correlating with immune-related genes, immune-inhibitory genes, and immune cell infiltration (41). However, these associations varied substantially by cancer type, suggesting that GALNT7 may exert complex and context-dependent roles in cancer progression and immune regulation (41).

In our IHC analysis, Tn antigen levels were higher in GALNT7-High colorectal cancers than in GALNT7-Low colorectal cancers, with this association being most prominent in dMMR/MSI colorectal cancers and to a lesser extent in pMMR/MSS colorectal cancers. One possibility is that the upregulation of GALNT7 in dMMR/MSI colorectal cancers directly contributes to the increased Tn antigen expression. Indeed, in prostate cancer and melanoma cells, GALNT7 depletion resulted in decreased Tn antigen levels, as demonstrated by lectin microarray and flow cytometry analyses (39, 40). However, in this study, GALNT7 knockdown in dMMR/MSI colorectal cancer cell lines did not alter Tn antigen–binding lectin signals or cell-surface Tn antigen expression. These discrepancies between IHC and *in vitro* results, as well as between colorectal cancer and other cancers, may be attributed to the complex and context-dependent regulation of O-glycosylation. The initial step of O-glycosylation, which forms the Tn antigen, is mediated by a coordinated and occasionally competitive interplay among 20 GALNT isoenzymes. These enzymes exhibit overlapping but distinct substrate specificities and catalyze the transfer of GalNAc to Ser/Thr residues on substrate proteins (42). Thus, knocking down a single GALNT, such as GALNT7, may not produce a strong phenotype because of functional redundancy among these isoenzymes. Although GALNT7 was not directly responsible for the regulation of Tn antigen at least *in vitro*, the dysregulation of multiple GALNT isoenzymes may contribute to aberrant Tn antigen synthesis in dMMR/MSI colorectal cancer cells. Beyond GALNT isoenzymes, it is important to note that the upregulation of Tn antigen is primarily driven by inactivation of *Cosmc*, which occurs through loss-of-function mutations or epigenetic silencing in colorectal cancer (43, 44). The repetitive DNA sequences in the *Cosmc* gene seem to be highly susceptible to MSI (43), suggesting that *Cosmc* inactivation is a major contributor to Tn antigen upregulation in dMMR/MSI colorectal cancers.

This study has several limitations. Despite the large number of colorectal cancer samples analyzed, our findings were derived primarily from retrospective datasets. Therefore, prospective validation, particularly for the prognostic value of GALNT7 in independent dMMR/MSI colorectal cancer cohorts, is required. Although our analyses consistently demonstrated a favorable prognostic impact of high GALNT7 expression in dMMR/MSI colorectal cancers across multiple cohorts, the observational design precludes a definitive mechanistic explanation for this dMMR/MSI-restricted effect. In addition, future studies should assess whether GALNT7 expression influences the response to ICIs in patients with dMMR/MSI CRC. Although we observed a consistent inverse correlation between GALNT7 and PD-L1 expression, the direct molecular mechanism by which GALNT7 regulates PD-L1 expression requires further elucidation. Given that GALNT7 modulates immune-related pathways in multiple cancer types, broader investigations into its role across different tumor contexts may uncover novel insights into its potential as a therapeutic target for immunotherapy.

In conclusion, we identified a set of glycosyltransferases, including GALNT7, that display differential expression depending on MMR/MSI status. Among them, GALNT7 effectively stratified dMMR/MSI colorectal cancers into two molecularly distinct subsets, characterized by differential tumor cell PD-L1 expression and distinct survival outcomes. These findings underscore the potential of GALNT7 as a prognostic biomarker and its relevance in refining treatment strategies, particularly in the context of immunotherapy.

## Supplementary Material

Supplementary Data Supplementary Table S1-S7, Suuplemntary Figure S1-S16
