# Supplementary material for: GALNT7 Stratifies dMMR/MSI Colorectal Cancer into Distinct Molecular Subsets Associated with Prognosis and PD-L1 Expression
Source: Cancer Res Commun. 2025 Sep 5;5(9):1530–40. doi: 10.1158/2767-9764.CRC-25-0270 (PMC12412012; doi:10.1158/2767-9764.CRC-25-0270)
Supplement: Supplementary Data — Supplementary Table S1-S7, Suuplemntary Figure S1-S16 [file crc-25-0270_supplementary_data_suppst1-st7_sf1-sf16.pdf]

Supplementary Table S1. List of cohorts used in this study

Cohorts	Data sources	Platforms	Total (n=4325)	MSI/MMR status			References
				MSI/ dMMR (n=662)	MSS/ pMMR (n=3483)	unknown (n=180)	
TCGA	cBioPortal	Illumina HiSeq 2000	592	76	450	66	(1)
CCLE	cBioPortal	Illumina HiSeq 2000/2500	54	20	34	0	(2)
SMC (GSE132465)	GEO	Illumina HiSeq 4000	23	4 (1813 single epithelial cells)	19 (15656 single epithelial cells)	0	(3)
AC-ICAM	cBioPortal	Illumina HiSeq 4000	348	57	224	67	(4)
GSE39582	GEO	Affymetrix HG-U133+2.0	566	75	444	47	(5)
GSE26682	GEO	Affymetrix HG-U133+2.0	300	35	265	0	(6)
GSE75315	GEO	Affymetrix Human Exon 1.0ST	206	24	182	0	(7)
GSE41258	GEO	Affymetrix HG-U133A	168	35	133	0	(8)
GSE13294	GEO	Affymetrix HG-U133+2.0	155	78	77	0	(9)
GSE24551	GEO	Affymetrix Human Exon 1.0ST	147	21	126	0	(10)
GSE33113	GEO	Affymetrix HG-U133+2.0	90	25	65	0	(11)
GSE42284	GEO	Agilent Homo sapiens 37K DiscoverPrint_19742	90	15	75	0	(12)
GSE143985	GEO	Affymetrix HG-U133+2.0	90	5	85	0	(13)
GSE4554	GEO	Affymetrix HG-U133+2.0	84	33	51	0	(14)
GSE13067	GEO	Affymetrix HG-U133+2.0	74	11	63	0	(9)
GSE39084	GEO	Affymetrix HG-U133+2.0	70	16	54	0	(15)

GSE35896	GEO	Affymetrix HG-U133+2.0	61	5	56	0	(16)
GSE18088	GEO	Affymetrix HG-U133+2.0	53	19	34	0	(17)
FOCUS-FFPE (GSE156915)	GEO	Almac Diagnostics Custom Xcel array	343	13	330	0	(18)
CPTAC-protein	cBioPortal	mass spectrometry	86	20	66	0	(19)
CPTAC-RNA	cBioPortal	Illumina HiSeq 4000	106	24	82	0	(19)
FMU-IHC	-	immunohistochemistry	619	51	568	0	-

References for Supplementary Table 1

1. Cancer Genome Atlas N. Comprehensive molecular characterization of human colon and rectal cancer. *Nature* 2012;**487**(7407):330-7 doi 10.1038/nature11252.
2. Ghandi M, Huang FW, Jane-Valbuena J, Kryukov GV, Lo CC, McDonald ER, 3rd*, et al.* Next-generation characterization of the Cancer Cell Line Encyclopedia. *Nature* 2019;**569**(7757):503-8 doi 10.1038/s41586-019-1186-3.
3. Lee HO, Hong Y, Etlioglu HE, Cho YB, Pomella V, Van den Bosch B*, et al.* Lineage-dependent gene expression programs influence the immune landscape of colorectal cancer. *Nat Genet* 2020;**52**(6):594-603 doi 10.1038/s41588-020-0636-z.
4. Roelands J, Kuppen PJK, Ahmed EI, Mall R, Masoodi T, Singh P*, et al.* An integrated tumor, immune and microbiome atlas of colon cancer. *Nat Med* 2023;**29**(5):1273-86 doi 10.1038/s41591-023-02324-5.
5. Marisa L, de Reyniès A, Duval A, Selves J, Gaub MP, Vescovo L*, et al.* Gene expression classification of colon cancer into molecular subtypes: characterization, validation, and prognostic value. *PLoS Med* 2013;**10**(5):e1001453 doi 10.1371/journal.pmed.1001453.
6. Vilar E, Bartnik CM, Stenzel SL, Raskin L, Ahn J, Moreno V*, et al.* MRE11 deficiency increases sensitivity to poly(ADP-ribose) polymerase inhibition in microsatellite unstable colorectal cancers. *Cancer Res* 2011;**71**(7):2632-42 doi 10.1158/0008-5472.CAN-10-1120.
7. Barras D, Missiaglia E, Wirapati P, Sieber OM, Jorissen RN, Love C*, et al.* BRAF V600E Mutant Colorectal Cancer Subtypes Based on Gene Expression. *Clin Cancer Res* 2017;**23**(1):104-15 doi 10.1158/1078-0432.CCR-16-0140.
8. Sheffer M, Bacolod MD, Zuk O, Giardina SF, Pincas H, Barany F*, et al.* Association of survival and disease progression with chromosomal instability: a genomic exploration of colorectal cancer. *Proc Natl Acad Sci U S A* 2009;**106**(17):7131-6 doi 10.1073/pnas.0902232106.
9. Jorissen RN, Lipton L, Gibbs P, Chapman M, Desai J, Jones IT*, et al.* DNA copy-number alterations underlie gene expression differences between microsatellite stable and unstable colorectal cancers. *Clin Cancer Res* 2008;**14**(24):8061-9 doi 10.1158/1078-0432.CCR-08-1431.

10. Sveen A, Agesen TH, Nesbakken A, Rognum TO, Lothe RA, Skotheim RI. Transcriptome instability in colorectal cancer identified by exon microarray analyses: Associations with splicing factor expression levels and patient survival. *Genome Med* 2011;**3**(5):32 doi 10.1186/gm248.

11. de Sousa E Melo F, Colak S, Buikhuisen J, Koster J, Cameron K, de Jong JH*, et al.* Methylation of cancer-stem-cell-associated Wnt target genes predicts poor prognosis in colorectal cancer patients. *Cell Stem Cell* 2011;**9**(5):476-85 doi 10.1016/j.stem.2011.10.008.

12. Roepman P, Schlicker A, Tabernero J, Majewski I, Tian S, Moreno V*, et al.* Colorectal cancer intrinsic subtypes predict chemotherapy benefit, deficient mismatch repair and epithelial-to-mesenchymal transition. *Int J Cancer* 2014;**134**(3):552-62 doi 10.1002/ijc.28387.

13. Shinto E, Yoshida Y, Kajiwara Y, Okamoto K, Mochizuki S, Yamadera M*, et al.* Clinical Significance of a Gene Signature Generated from Tumor Budding Grade in Colon Cancer. *Ann Surg Oncol* 2020;**27**(10):4044-54 doi 10.1245/s10434-020-08498-3.

14. Watanabe T, Kobunai T, Toda E, Yamamoto Y, Kanazawa T, Kazama Y*, et al.* Distal colorectal cancers with microsatellite instability (MSI) display distinct gene expression profiles that are different from proximal MSI cancers. *Cancer Res* 2006;**66**(20):9804-8 doi 10.1158/0008-5472.CAN-06-1163.

15. Kirzin S, Marisa L, Guimbaud R, De Reynies A, Legrain M, Laurent-Puig P*, et al.* Sporadic early-onset colorectal cancer is a specific sub-type of cancer: a morphological, molecular and genetics study. *PLoS One* 2014;**9**(8):e103159 doi 10.1371/journal.pone.0103159.

16. Schlicker A, Beran G, Chresta CM, McWalter G, Pritchard A, Weston S*, et al.* Subtypes of primary colorectal tumors correlate with response to targeted treatment in colorectal cell lines. *BMC Med Genomics* 2012;**5**:66 doi 10.1186/1755-8794-5-66.

17. Gröne J, Lenze D, Jurinovic V, Hummel M, Seidel H, Leder G*, et al.* Molecular profiles and clinical outcome of stage UICC II colon cancer patients. *Int J Colorectal Dis* 2011;**26**(7):847-58 doi 10.1007/s00384-011-1176-x.

18. Malla SB, Fisher DJ, Domingo E, Blake A, Hassanieh S, Redmond KL*, et al.* In-depth Clinical and Biological Exploration of DNA Damage Immune Response as a Biomarker for Oxaliplatin Use in Colorectal Cancer. *Clin Cancer Res* 2021;**27**(1):288-300 doi 10.1158/1078-0432.CCR-20-3237.

19. Vasaikar S, Huang C, Wang X, Petyuk VA, Savage SR, Wen B*, et al.* Proteogenomic Analysis of Human Colon Cancer Reveals New Therapeutic Opportunities. *Cell* 2019;**177**(4):1035-49 e19 doi 10.1016/j.cell.2019.03.030.

Supplementary Table S2. List of glycosyltransferase genes in KEGG gene sets

Gene Set	NCBI Gene Id	Gene Symbol	Gene Description
KEGG_N_GLYCAN_BIOSYNTHESIS	10195	ALG3	ALG3 alpha-1,3- mannosyltransferase
	10905	MAN1A2	mannosidase alpha class 1A member 2
	11253	MAN1B1	mannosidase alpha class 1B member 1
	11282	MGAT4B	alpha-1,3-mannosyl-glycoprotein 4-beta-N-acetylglucosaminyltransferase B
	11320	MGAT4A	alpha-1,3-mannosyl-glycoprotein 4-beta-N-acetylglucosaminyltransferase A
	144245	ALG10B	ALG10 alpha-1,2-glucosyltransferase B
	146664	MGAT5B	alpha-1,6-mannosylglycoprotein 6-beta-N-acetylglucosaminyltransferase B
	1603	DAD1	defender against cell death 1
	1650	DDOST	dolichyl-diphosphooligosaccharide--protein glycosyltransferase non-catalytic subunit
	1798	DPAGT1	dolichyl-phosphate N-acetylglucosaminephosphotransferase 1
	199857	ALG14	ALG14 UDP-N-acetylglucosaminyltransferase subunit
	201595	STT3B	STT3 oligosaccharyltransferase complex catalytic subunit B
	23193	GANAB	glucosidase II alpha subunit
	2530	FUT8	fucosyltransferase 8
	2683	B4GALT1	beta-1,4-galactosyltransferase 1
	29880	ALG5	ALG5 dolichyl-phosphate beta-glucosyltransferase
	29929	ALG6	ALG6 alpha-1,3-glucosyltransferase
	3703	STT3A	STT3 oligosaccharyltransferase complex catalytic subunit A
	4121	MAN1A1	mannosidase alpha class 1A member 1
	4122	MAN2A2	mannosidase alpha class 2A member 2
	4124	MAN2A1	mannosidase alpha class 2A member 1
	4245	MGAT1	alpha-1,3-mannosyl-glycoprotein 2-beta-N-acetylglucosaminyltransferase
	4247	MGAT2	alpha-1,6-mannosyl-glycoprotein 2-beta-N-acetylglucosaminyltransferase
	4248	MGAT3	beta-1,4-mannosyl-glycoprotein 4-beta-N-acetylglucosaminyltransferase
	4249	MGAT5	alpha-1,6-mannosylglycoprotein 6-beta-N-acetylglucosaminyltransferase
	440138	ALG11	ALG11 alpha-1,2-mannosyltransferase
	54344	DPM3	dolichyl-phosphate mannosyltransferase subunit 3, regulatory
	56052	ALG1	ALG1 chitobiosyldiphosphodolichol beta-mannosyltransferase
	57134	MAN1C1	mannosidase alpha class 1C member 1
	57171	DOLPP1	dolichyldiphosphatase 1
	6184	RPN1	ribophorin I
	6185	RPN2	ribophorin II
	6480	ST6GAL1	ST6 beta-galactoside alpha-2,6-sialyltransferase 1
	7841	MOGS	mannosyl-oligosaccharide glucosidase
	79053	ALG8	ALG8 alpha-1,3-glucosyltransferase
	79087	ALG12	ALG12 alpha-1,6-mannosyltransferase
	79796	ALG9	ALG9 alpha-1,2-mannosyltransferase
	79868	ALG13	ALG13 UDP-N-acetylglucosaminyltransferase subunit
	7991	TUSC3	tumor suppressor candidate 3
	84920	ALG10	ALG10 alpha-1,2-glucosyltransferase
	85365	ALG2	ALG2 alpha-1,3/1,6-mannosyltransferase
	8703	B4GALT3	beta-1,4-galactosyltransferase 3
	8704	B4GALT2	beta-1,4-galactosyltransferase 2
	8813	DPM1	dolichyl-phosphate mannosyltransferase subunit 1, catalytic
	8818	DPM2	dolichyl-phosphate mannosyltransferase subunit 2, regulatory
	91869	RFT1	RFT1 homolog
KEGG_O_GLYCAN_BIOSYNTHESIS	11226	GALNT6	polypeptide N-acetylgalactosaminyltransferase 6
	11227	GALNT5	polypeptide N-acetylgalactosaminyltransferase 5
	114805	GALNT13	polypeptide N-acetylgalactosaminyltransferase 13
	117248	GALNT15	polypeptide N-acetylgalactosaminyltransferase 15
	168391	GALNTL5	polypeptide N-acetylgalactosaminyltransferase like 5
	192134	B3GNT6	UDP-GlcNAc:betaGal beta-1,3-N-acetylglucosaminyltransferase 6
	2589	GALNT1	polypeptide N-acetylgalactosaminyltransferase 1
	2590	GALNT2	polypeptide N-acetylgalactosaminyltransferase 2
	2591	GALNT3	polypeptide N-acetylgalactosaminyltransferase 3
	26290	GALNT8	polypeptide N-acetylgalactosaminyltransferase 8
	2650	GCNT1	glucosaminyl (N-acetyl) transferase 1
	29071	C1GALT1C1	C1GALT1 specific chaperone 1
	374378	GALNT18	polypeptide N-acetylgalactosaminyltransferase 18
	442117	GALNTL6	polypeptide N-acetylgalactosaminyltransferase like 6

	50614	GALNT9	polypeptide N-acetylgalactosaminyltransferase 9
	51301	GCNT4	glucosaminyl (N-acetyl) transferase 4
	51809	GALNT7	polypeptide N-acetylgalactosaminyltransferase 7
	55568	GALNT10	polypeptide N-acetylgalactosaminyltransferase 10
	55808	ST6GALNAC1	ST6 N-acetylgalactosaminide alpha-2,6-sialyltransferase 1
	56913	C1GALT1	core 1 synthase, glycoprotein-N-acetylgalactosamine 3-beta-galactosyltransferase 1
	57452	GALNT16	polypeptide N-acetylgalactosaminyltransferase 16
	63917	GALNT11	polypeptide N-acetylgalactosaminyltransferase 11
	64409	GALNT17	polypeptide N-acetylgalactosaminyltransferase 17
	6482	ST3GAL1	ST3 beta-galactoside alpha-2,3-sialyltransferase 1
	6483	ST3GAL2	ST3 beta-galactoside alpha-2,3-sialyltransferase 2
	79623	GALNT14	polypeptide N-acetylgalactosaminyltransferase 14
	79695	GALNT12	polypeptide N-acetylgalactosaminyltransferase 12
	8693	GALNT4	polypeptide N-acetylgalactosaminyltransferase 4
	9245	GCNT3	glucosaminyl (N-acetyl) transferase 3, mucin type
	9334	B4GALT5	beta-1,4-galactosyltransferase 5
KEGG_GLYCOSPHINGOLIPID_BIOSYNTHESIS_GANGLIO_SERIES	256435	ST6GALNAC3	ST6 N-acetylgalactosaminide alpha-2,6-sialyltransferase 3
	2583	B4GALNT1	beta-1,4-N-acetyl-galactosaminyltransferase 1
	27090	ST6GALNAC4	ST6 N-acetylgalactosaminide alpha-2,6-sialyltransferase 4
	2720	GLB1	galactosidase beta 1
	29906	ST8SIA5	ST8 alpha-N-acetyl-neuraminide alpha-2,8-sialyltransferase 5
	3073	HEXA	hexosaminidase subunit alpha
	3074	HEXB	hexosaminidase subunit beta
	30815	ST6GALNAC6	ST6 N-acetylgalactosaminide alpha-2,6-sialyltransferase 6
	6482	ST3GAL1	ST3 beta-galactoside alpha-2,3-sialyltransferase 1
	6483	ST3GAL2	ST3 beta-galactoside alpha-2,3-sialyltransferase 2
	6489	ST8SIA1	ST8 alpha-N-acetyl-neuraminide alpha-2,8-sialyltransferase 1
	81849	ST6GALNAC5	ST6 N-acetylgalactosaminide alpha-2,6-sialyltransferase 5
	8705	B3GALT4	beta-1,3-galactosyltransferase 4
	8869	ST3GAL5	ST3 beta-galactoside alpha-2,3-sialyltransferase 5
	9197	SLC33A1	solute carrier family 33 member 1
KEGG_GLYCOSPHINGOLIPID_BIOSYNTHESIS_GLOBO_SERIES	10317	B3GALT5	beta-1,3-galactosyltransferase 5
	10690	FUT9	fucosyltransferase 9
	2523	FUT1	fucosyltransferase 1 (H blood group)
	2524	FUT2	fucosyltransferase 2
	26301	GBGT1	globoside alpha-1,3-N-acetylgalactosaminyltransferase 1 (FORS blood group)
	2717	GLA	galactosidase alpha
	3073	HEXA	hexosaminidase subunit alpha
	3074	HEXB	hexosaminidase subunit beta
	4668	NAGA	alpha-N-acetylgalactosaminidase
	53947	A4GALT	alpha 1,4-galactosyltransferase (P1PK blood group)
	6482	ST3GAL1	ST3 beta-galactoside alpha-2,3-sialyltransferase 1
	6483	ST3GAL2	ST3 beta-galactoside alpha-2,3-sialyltransferase 2
	6489	ST8SIA1	ST8 alpha-N-acetyl-neuraminide alpha-2,8-sialyltransferase 1
	8706	B3GALNT1	beta-1,3-N-acetylgalactosaminyltransferase 1 (Globoside blood group)
KEGG_GLYCOSPHINGOLIPID_BIOSYNTHESIS_LACTO_AND_NEOLACTO_SERIES	10317	B3GALT5	beta-1,3-galactosyltransferase 5
	10331	B3GNT3	UDP-GlcNAc:betaGal beta-1,3-N-acetylglucosaminyltransferase 3
	10402	ST3GAL6	ST3 beta-galactoside alpha-2,3-sialyltransferase 6
	10678	B3GNT2	UDP-GlcNAc:betaGal beta-1,3-N-acetylglucosaminyltransferase 2
	10690	FUT9	fucosyltransferase 9
	11041	B4GAT1	beta-1,4-glucuronyltransferase 1
	2523	FUT1	fucosyltransferase 1 (H blood group)
	2524	FUT2	fucosyltransferase 2
	2525	FUT3	fucosyltransferase 3 (Lewis blood group)
	2526	FUT4	fucosyltransferase 4
	2527	FUT5	fucosyltransferase 5
	2528	FUT6	fucosyltransferase 6
	2529	FUT7	fucosyltransferase 7
	2651	GCNT2	glucosaminyl (N-acetyl) transferase 2 (I blood group)
	2683	B4GALT1	beta-1,4-galactosyltransferase 1
	28	ABO	ABO, alpha 1-3-N-acetylgalactosaminyltransferase and alpha 1-3-galactosyltransferase
	6484	ST3GAL4	ST3 beta-galactoside alpha-2,3-sialyltransferase 4
	6487	ST3GAL3	ST3 beta-galactoside alpha-2,3-sialyltransferase 3

	6489	ST8SIA1	ST8 alpha-N-acetyl-neuraminide alpha-2,8-sialyltransferase 1
	79369	B3GNT4	UDP-GlcNAc:betaGal beta-1,3-N-acetylglucosaminyltransferase 4
	84002	B3GNT5	UDP-GlcNAc:betaGal beta-1,3-N-acetylglucosaminyltransferase 5
	8702	B4GALT4	beta-1,4-galactosyltransferase 4
	8703	B4GALT3	beta-1,4-galactosyltransferase 3
	8704	B4GALT2	beta-1,4-galactosyltransferase 2
	8707	B3GALT2	beta-1,3-galactosyltransferase 2
	8708	B3GALT1	beta-1,3-galactosyltransferase 1
KEGG_GLYCOSAMINOGLYCAN_BIOSYNTHESIS_CHONDROITIN_SULFATE	10090	UST	uronyl 2-sulfotransferase
	11285	B4GALT7	beta-1,4-galactosyltransferase 7
	113189	CHST14	carbohydrate sulfotransferase 14
	126792	B3GALT6	beta-1,3-galactosyltransferase 6
	135152	B3GAT2	beta-1,3-glucuronyltransferase 2
	166012	CHST13	carbohydrate sulfotransferase 13
	22856	CHSY1	chondroitin sulfate synthase 1
	26229	B3GAT3	beta-1,3-glucuronyltransferase 3
	27087	B3GAT1	beta-1,3-glucuronyltransferase 1
	29940	DSE	dermatan sulfate epimerase
	337876	CHSY3	chondroitin sulfate synthase 3
	50515	CHST11	carbohydrate sulfotransferase 11
	51363	CHST15	carbohydrate sulfotransferase 15
	54480	CHPF2	chondroitin polymerizing factor 2
	55454	CSGALNACT2	chondroitin sulfate N-acetylgalactosaminyltransferase 2
	55501	CHST12	carbohydrate sulfotransferase 12
	55790	CSGALNACT1	chondroitin sulfate N-acetylgalactosaminyltransferase 1
	56548	CHST7	carbohydrate sulfotransferase 7
	64131	XYLT1	xylosyltransferase 1
	64132	XYLT2	xylosyltransferase 2
	79586	CHPF	chondroitin polymerizing factor
	9469	CHST3	carbohydrate sulfotransferase 3
KEGG_GLYCOSAMINOGLYCAN_BIOSYNTHESIS_HEPARAN_SULFATE	11285	B4GALT7	beta-1,4-galactosyltransferase 7
	126792	B3GALT6	beta-1,3-galactosyltransferase 6
	135152	B3GAT2	beta-1,3-glucuronyltransferase 2
	2131	EXT1	exostosin glycosyltransferase 1
	2132	EXT2	exostosin glycosyltransferase 2
	2134	EXTL1	exostosin like glycosyltransferase 1
	2135	EXTL2	exostosin like glycosyltransferase 2
	2137	EXTL3	exostosin like glycosyltransferase 3
	222537	HS3ST5	heparan sulfate-glucosamine 3-sulfotransferase 5
	26035	GLCE	glucuronic acid epimerase
	26229	B3GAT3	beta-1,3-glucuronyltransferase 3
	266722	HS6ST3	heparan sulfate 6-O-sulfotransferase 3
	27087	B3GAT1	beta-1,3-glucuronyltransferase 1
	3340	NDST1	N-deacetylase and N-sulfotransferase 1
	64131	XYLT1	xylosyltransferase 1
	64132	XYLT2	xylosyltransferase 2
	64579	NDST4	N-deacetylase and N-sulfotransferase 4
	8509	NDST2	N-deacetylase and N-sulfotransferase 2
	90161	HS6ST2	heparan sulfate 6-O-sulfotransferase 2
	9348	NDST3	N-deacetylase and N-sulfotransferase 3
	9394	HS6ST1	heparan sulfate 6-O-sulfotransferase 1
	9653	HS2ST1	heparan sulfate 2-O-sulfotransferase 1
	9953	HS3ST3B1	heparan sulfate-glucosamine 3-sulfotransferase 3B1
	9955	HS3ST3A1	heparan sulfate-glucosamine 3-sulfotransferase 3A1
	9956	HS3ST2	heparan sulfate-glucosamine 3-sulfotransferase 2
	9957	HS3ST1	heparan sulfate-glucosamine 3-sulfotransferase 1
KEGG_GLYCOSAMINOGLYCAN_BIOSYNTHESIS_KERATAN_SULFATE	10164	CHST4	carbohydrate sulfotransferase 4
	10678	B3GNT2	UDP-GlcNAc:betaGal beta-1,3-N-acetylglucosaminyltransferase 2
	11041	B4GAT1	beta-1,4-glucuronyltransferase 1
	2530	FUT8	fucosyltransferase 8
	2683	B4GALT1	beta-1,4-galactosyltransferase 1
	4166	CHST6	carbohydrate sulfotransferase 6
	6482	ST3GAL1	ST3 beta-galactoside alpha-2,3-sialyltransferase 1

	6483	ST3GAL2	ST3 beta-galactoside alpha-2,3-sialyltransferase 2
	6487	ST3GAL3	ST3 beta-galactoside alpha-2,3-sialyltransferase 3
	8534	CHST1	carbohydrate sulfotransferase 1
	8702	B4GALT4	beta-1,4-galactosyltransferase 4
	8703	B4GALT3	beta-1,4-galactosyltransferase 3
	8704	B4GALT2	beta-1,4-galactosyltransferase 2
	93010	B3GNT7	UDP-GlcNAc:betaGal beta-1,3-N-acetylglucosaminyltransferase 7
	9435	CHST2	carbohydrate sulfotransferase 2
KEGG_GLYCOSAMINOGLYCAN_DEGRADATION	10855	HPSE	heparanase
	138050	HGSNAT	heparan-alpha-glucosaminide N-acetyltransferase
	23553	HYAL4	hyaluronidase 4
	2588	GALNS	galactosamine (N-acetyl)-6-sulfatase
	2720	GLB1	galactosidase beta 1
	2799	GNS	glucosamine (N-acetyl)-6-sulfatase
	2990	GUSB	glucuronidase beta
	3073	HEXA	hexosaminidase subunit alpha
	3074	HEXB	hexosaminidase subunit beta
	3373	HYAL1	hyaluronidase 1
	3423	IDS	iduronate 2-sulfatase
	3425	IDUA	alpha-L-iduronidase
	411	ARSB	arylsulfatase B
	4669	NAGLU	N-acetyl-alpha-glucosaminidase
	60495	HPSE2	heparanase 2 (inactive)
	6448	SGSH	N-sulfoglucosamine sulfohydrolase
	6677	SPAM1	sperm adhesion molecule 1
	8372	HYAL3	hyaluronidase 3
	8692	HYAL2	hyaluronidase 2
	9953	HS3ST3B1	heparan sulfate-glucosamine 3-sulfotransferase 3B1
	9955	HS3ST3A1	heparan sulfate-glucosamine 3-sulfotransferase 3A1
KEGG_OTHER_GLYCAN_DEGRADATION	10825	NEU3	neuraminidase 3
	129807	NEU4	neuraminidase 4
	175	AGA	aspartylglucosaminidase
	23324	MAN2B2	mannosidase alpha class 2B member 2
	2517	FUCA1	alpha-L-fucosidase 1
	2519	FUCA2	alpha-L-fucosidase 2
	2629	GBA1	glucosylceramidase beta 1
	2720	GLB1	galactosidase beta 1
	3073	HEXA	hexosaminidase subunit alpha
	3074	HEXB	hexosaminidase subunit beta
	4123	MAN2C1	mannosidase alpha class 2C member 1
	4125	MAN2B1	mannosidase alpha class 2B member 1
	4126	MANBA	mannosidase beta
	4758	NEU1	neuraminidase 1
	4759	NEU2	neuraminidase 2
	64772	ENGASE	endo-beta-N-acetylglucosaminidase

Supplementary Table S3. Differential gene expression analysis of glycosyltransferase genes between MSI and MSS CRC in bulk (TCGA), cell line (CCLE), and single-cell (SMC) RNA-seq datasets

	Bulk RNA-seq			Cell line RNA-seq			Single-cell RNA-seq			
Gene	MSI (n=76) z-score normalized expression	MSS (n=450) z-score normalized expression	log_2 fold change between MSI and MSS	MSI (n=20) z-score normalized expression	MSS (n=34) z-score normalized expression	log_2 fold change between MSI and MSS	percent expresed by tumor epithelial cells	MSI (1813 cells) z-score normalized expression	MSS (15656 cells) z-score normalized expression	log_2 fold change between MSI and MSS
ST6GAL1	-0.67	0.14	-1.01	-0.15	0.09	-0.47	18.47	-0.13	0.01	-0.58
GALNT6	-0.64	0.10	-0.69	-0.44	0.26	-0.85	34.50	-0.30	0.03	-1.10
HPSE	1.54	-0.22	1.47	0.73	-0.43	1.81	5.60	0.78	-0.09	3.07
GALNT1	0.89	-0.12	0.57	0.64	-0.37	0.80	49.56	0.29	-0.03	0.53
GALNT7	0.69	-0.09	0.57	0.29	-0.17	0.45	33.50	0.18	-0.02	0.43
A4GALT	0.17	0.00	0.23	-0.27	0.16	-1.71	1.70	-0.08	0.01	-1.99
ABO	-0.33	0.03	-0.46	-0.10	0.06	-0.26	23.13	0.13	-0.02	0.41
AGA	-0.54	0.12	-0.44	0.00	0.00	0.01	16.78	0.11	-0.01	0.41
ALG1	-0.19	0.07	-0.12	-0.03	0.02	-0.03	22.92	-0.09	0.01	-0.36
ALG10	0.22	-0.01	0.14	0.15	-0.09	0.15	3.69	0.01	0.00	0.05
ALG10B	-0.43	0.10	-0.50	-0.28	0.16	-0.54	2.72	-0.03	0.00	-0.37
ALG11	-0.02	-0.03	0.01	-0.16	0.09	-0.19	3.90	0.09	-0.01	0.73
ALG12	0.18	0.00	0.08	0.46	-0.27	0.38	14.32	0.16	-0.02	0.61
ALG13	0.03	-0.01	0.02	0.01	-0.01	0.01	44.82	0.37	-0.04	0.68
ALG14	-0.34	0.05	-0.23	-0.15	0.09	-0.16	30.51	-0.08	0.01	-0.23
ALG2	0.16	-0.01	0.07	0.06	-0.03	0.03	25.88	0.07	-0.01	0.21
ALG3	-0.45	0.09	-0.38	-0.43	0.25	-0.37	51.43	0.03	0.00	0.06
ALG5	-0.35	0.07	-0.34	-0.18	0.10	-0.23	61.25	-0.12	0.01	-0.20
ALG6	0.26	-0.02	0.16	0.51	-0.30	0.44	13.00	0.05	-0.01	0.22
ALG8	-0.40	0.07	-0.27	-0.43	0.25	-0.47	20.57	0.02	0.00	0.06
ALG9	0.23	-0.05	0.09	-0.07	0.04	-0.07	3.69	0.03	0.00	0.26
ARSB	0.32	-0.02	0.27	-0.05	0.03	-0.16	2.83	0.04	0.00	0.35
B3GALNT1	0.59	-0.08	0.73	0.36	-0.21	1.72	2.14	0.02	0.00	0.23
B3GALNT2	0.32	-0.08	0.16	0.66	-0.39	0.47	17.99	-0.06	0.01	-0.27
B3GALT1	-0.15	-0.01	-0.35	0.22	-0.13	1.43	1.14	-0.04	0.00	-0.76
B3GALT2	0.03	0.02	0.05	-0.16	0.10	-2.20	0.19	-0.01	0.00	-0.43
B3GALT4	0.05	0.03	0.01	-0.30	0.17	-0.63	9.81	0.25	-0.03	1.11
B3GALT5	0.27	-0.02	0.57	0.11	-0.07	0.36	5.08	0.04	0.00	0.32
B3GALT6	0.04	0.02	0.01	-0.21	0.13	-0.20	17.10	0.02	0.00	0.07
B3GAT1	0.20	-0.02	0.70	-0.03	0.02	-0.13	0.02	0.08	-0.01	4.14
B3GAT2	-0.25	-0.01	-0.30	0.00	0.00	0.03	6.61	-0.10	0.01	-1.02
B3GAT3	0.14	-0.01	0.08	-0.14	0.08	-0.11	35.53	0.33	-0.04	0.72
B3GNT2	0.34	-0.04	0.20	-0.36	0.21	-0.49	27.32	-0.07	0.01	-0.24
B3GNT3	-0.49	0.10	-0.41	-0.26	0.15	-0.52	42.82	-0.15	0.02	-0.38
B3GNT4	1.13	-0.19	1.70	0.47	-0.28	1.40	1.20	0.36	-0.04	3.04
B3GNT5	0.22	-0.02	0.14	-0.35	0.21	-0.53	43.85	-0.19	0.02	-0.50
B3GNT6	0.13	-0.02	0.49	-0.07	0.04	-0.44	1.32	-0.03	0.00	-0.61
B3GNT7	-0.04	0.03	-0.19	0.10	-0.06	0.56	13.06	0.07	-0.01	0.34
B4GALNT1	0.01	0.01	0.00	-0.02	0.01	-0.09	0.18	0.12	-0.01	2.58
B4GALT1	0.30	0.01	0.14	-0.21	0.12	-0.29	39.39	0.05	-0.01	0.12
B4GALT2	0.16	-0.02	0.09	0.26	-0.16	0.21	20.61	0.00	0.00	0.01
B4GALT3	-0.18	0.05	-0.12	0.00	0.00	0.00	38.54	-0.14	0.02	-0.37
B4GALT4	0.13	-0.02	0.08	-0.39	0.23	-0.42	27.47	0.31	-0.04	0.80
B4GALT5	-0.33	0.05	-0.19	-0.25	0.15	-0.41	51.97	-0.46	0.05	-1.26
B4GALT7	-0.24	0.06	-0.24	-0.13	0.08	-0.11	26.38	0.10	-0.01	0.30
B4GAT1	0.14	0.02	0.06	-0.26	0.15	-0.38	6.02	0.12	-0.01	0.74
C1GALT1	0.01	0.02	-0.01	-0.15	0.09	-0.17	38.90	0.08	-0.01	0.19
C1GALT1C1	-0.41	0.08	-0.31	-0.22	0.13	-0.22	32.53	-0.07	0.01	-0.21
CHPF	0.30	0.00	0.23	-0.25	0.15	-0.41	50.38	0.27	-0.03	0.48
CHPF2	-0.23	0.05	-0.13	-0.22	0.13	-0.24	18.83	0.10	-0.01	0.34
CHST1	0.05	-0.01	0.10	-0.19	0.11	-3.48	0.05	0.06	-0.01	2.78
CHST11	0.31	-0.04	0.47	-0.33	0.19	-2.97	0.90	0.02	0.00	0.28
CHST12	0.01	0.02	-0.01	-0.05	0.03	-0.06	14.54	0.01	0.00	0.03
CHST13	-0.39	0.06	-1.55	-0.35	0.21	-1.48	3.34	0.06	-0.01	0.50
CHST14	0.13	0.02	0.07	-0.29	0.17	-0.48	3.39	0.09	-0.01	0.70
CHST15	0.58	-0.07	0.76	0.18	-0.10	0.70	2.35	0.18	-0.02	1.49
CHST2	0.14	0.00	0.15	-0.04	0.02	-0.43	0.31	0.04	0.00	1.03
CHST3	0.12	-0.01	0.15	0.20	-0.12	0.67	1.50	0.15	-0.02	1.48
CHST4	0.52	-0.09	1.66	0.29	-0.17	1.27	1.01	-0.02	0.00	-0.38
CHST6	0.75	-0.12	1.68	0.26	-0.16	0.94	0.62	0.12	-0.01	1.90
CHST7	-0.10	0.00	-0.10	-0.20	0.12	-0.92	3.11	0.02	0.00	0.18
CHSY1	0.67	-0.09	0.43	0.52	-0.31	0.56	9.62	0.01	0.00	0.07
CHSY3	-0.02	-0.01	-0.01	0.31	-0.18	1.35	0.09	0.05	-0.01	1.77
CSGALNACT1	-0.04	0.03	-0.10	0.11	-0.06	0.33	1.55	-0.10	0.01	-4.93
DAD1	0.42	-0.02	0.23	-0.20	0.12	-0.14	80.23	0.74	-0.09	0.68

DDOST	0.47	-0.05	0.21	0.30	-0.17	0.16	70.83	0.05	-0.01	0.06
DOLPP1	-0.21	0.06	-0.15	-0.42	0.24	-0.34	9.91	0.06	-0.01	0.31
DPAGT1	-0.10	0.00	-0.04	-0.39	0.23	-0.30	24.70	0.02	0.00	0.07
DPM1	-0.81	0.13	-0.79	-0.34	0.20	-0.37	65.92	-0.40	0.05	-0.75
DPM2	-0.15	0.02	-0.13	-0.05	0.03	-0.04	53.32	0.09	-0.01	0.16
DPM3	0.00	0.01	-0.02	0.45	-0.27	0.65	58.33	0.17	-0.02	0.28
DSE	0.05	0.02	0.03	0.18	-0.11	0.47	3.41	0.29	-0.03	1.84
ENGASE	-0.86	0.09	-1.39	-0.34	0.20	-0.90	21.80	0.10	-0.01	0.34
EXT1	0.08	-0.01	0.04	0.02	-0.01	0.03	17.68	0.03	0.00	0.11
EXT2	-0.25	0.08	-0.12	-0.25	0.15	-0.25	23.35	-0.06	0.01	-0.22
EXTL1	-0.01	0.02	-0.10	-0.19	0.11	-0.61	0.10	0.01	0.00	0.68
EXTL2	0.16	-0.01	0.11	0.58	-0.34	0.85	9.88	0.09	-0.01	0.46
EXTL3	0.35	-0.06	0.25	0.27	-0.16	0.38	6.32	0.09	-0.01	0.55
FUCA1	0.15	0.00	0.09	0.08	-0.05	0.11	39.58	0.27	-0.03	0.57
FUCA2	0.10	-0.03	0.05	-0.08	0.05	-0.08	47.77	0.02	0.00	0.05
FUT1	0.21	-0.03	0.20	0.17	-0.10	0.27	5.16	0.06	-0.01	0.44
FUT2	0.04	-0.01	0.04	-0.13	0.08	-0.29	30.28	0.10	-0.01	0.28
FUT3	0.21	-0.05	0.19	0.36	-0.21	0.91	45.22	0.24	-0.03	0.48
FUT4	0.13	-0.04	0.11	na	na	na	27.80	-0.13	0.02	-0.45
FUT5	-0.02	-0.03	0.02	na	na	na	0.00	na	na	na
FUT6	-0.03	0.01	-0.03	0.17	-0.10	0.56	18.05	0.00	0.00	0.00
FUT7	-0.16	0.06	-0.59	-0.14	0.08	-0.74	1.16	-0.04	0.00	-0.92
FUT8	1.14	-0.16	0.91	-0.01	0.01	-0.02	22.32	0.18	-0.02	0.56
FUT9	0.16	-0.01	1.50	-0.10	0.06	-1.30	0.00	na	na	na
GALNS	-0.28	0.06	-0.23	-0.27	0.16	-0.31	15.53	0.01	0.00	0.04
GALNT10	-0.23	0.02	-0.16	-0.03	0.02	-0.04	16.72	0.01	0.00	0.05
GALNT11	-0.77	0.11	-0.44	-0.11	0.06	-0.14	22.45	-0.02	0.00	-0.08
GALNT12	0.49	-0.11	0.46	0.11	-0.07	0.18	23.95	0.04	0.00	0.14
GALNT13	0.47	-0.06	2.02	0.02	-0.01	0.21	0.24	0.13	-0.02	3.10
GALNT14	-0.03	-0.01	-0.07	-0.12	0.07	-0.89	0.38	0.26	-0.03	3.57
GALNT15	0.13	-0.01	0.32	0.19	-0.11	0.86	0.06	0.03	0.00	1.52
GALNT16	-0.22	0.07	-0.94	0.18	-0.11	1.06	0.24	-0.01	0.00	-0.29
GALNT18	0.23	-0.07	0.31	0.02	-0.01	0.06	5.95	-0.01	0.00	-0.07
GALNT2	-0.20	0.05	-0.12	-0.15	0.09	-0.19	28.60	-0.11	0.01	-0.37
GALNT3	0.36	-0.05	0.26	0.08	-0.05	0.12	52.89	-0.23	0.03	-0.51
GALNT4	-0.32	0.07	-0.44	na	na	na	0.26	-0.03	0.00	-3.53
GALNT5	0.75	-0.13	0.74	0.00	0.00	0.01	24.65	0.09	-0.01	0.27
GALNT8	0.13	-0.02	0.46	0.11	-0.06	0.37	3.73	0.20	-0.02	1.37
GALNT9	0.12	0.00	0.53	-0.08	0.05	-0.50	0.76	-0.04	0.01	-1.48
GANAB	0.41	-0.06	0.16	-0.03	0.01	-0.02	49.91	0.15	-0.02	0.27
GBA1	0.01	0.03	-0.01	-0.17	0.10	-0.19	15.29	0.19	-0.02	0.72
GBGT1	-0.53	0.10	-0.91	-0.25	0.15	-1.93	2.53	-0.11	0.01	-2.30
GCNT1	-0.04	0.01	-0.05	-0.05	0.03	-0.13	21.15	0.12	-0.01	0.40
GCNT2	0.21	-0.03	0.45	-0.23	0.13	-2.48	0.18	0.03	0.00	0.97
GCNT3	0.56	-0.09	1.03	0.03	-0.02	0.13	24.65	0.64	-0.07	1.53
GCNT4	-0.09	0.05	-0.29	-0.03	0.02	-0.16	0.22	-0.01	0.00	-0.35
GLA	-0.02	0.01	-0.02	-0.16	0.09	-0.16	28.52	0.01	0.00	0.04
GLB1	-0.48	0.11	-0.28	-0.25	0.15	-0.26	37.24	-0.04	0.00	-0.10
GLCE	-0.47	0.08	-0.46	-0.12	0.07	-0.17	37.27	-0.17	0.02	-0.49
GNS	0.70	-0.08	0.39	-0.13	0.08	-0.12	26.72	0.03	0.00	0.08
GUSB	-0.10	0.01	-0.07	-0.03	0.02	-0.03	43.63	0.01	0.00	0.02
HEXA	0.75	-0.08	0.40	-0.10	0.06	-0.12	37.92	0.17	-0.02	0.39
HEXB	0.00	0.04	-0.02	0.03	-0.02	0.03	66.90	0.04	-0.01	0.06
HGSNAT	-0.36	0.06	-0.27	0.06	-0.03	0.07	21.29	0.06	-0.01	0.20
HPSE2	-0.17	0.03	-1.46	0.14	-0.08	0.56	0.01	-0.01	0.00	na
HS2ST1	-0.15	0.06	-0.11	0.11	-0.06	0.09	21.17	-0.10	0.01	-0.43
HS3ST1	0.73	-0.08	0.76	-0.06	0.04	-0.16	13.99	0.08	-0.01	0.35
HS3ST2	0.30	-0.02	0.57	0.09	-0.05	0.63	0.02	-0.01	0.00	na
HS3ST3A1	0.01	0.00	0.02	-0.15	0.09	-4.54	0.05	0.00	0.00	0.24
HS3ST3B1	0.19	-0.03	0.53	-0.06	0.04	-0.34	0.50	0.12	-0.01	1.95
HS3ST5	-0.16	0.04	-1.29	-0.21	0.12	-3.76	0.22	-0.04	0.00	na
HS6ST1	0.05	-0.01	0.04	0.35	-0.20	0.57	10.64	-0.02	0.00	-0.13
HS6ST2	0.39	-0.06	0.60	0.18	-0.10	0.46	6.97	0.03	0.00	0.20
HS6ST3	0.00	-0.02	0.09	-0.11	0.06	-1.34	0.01	-0.01	0.00	na
HYAL1	0.46	-0.08	1.02	-0.16	0.09	-0.77	2.60	0.26	-0.03	1.92
HYAL2	-0.29	0.07	-0.30	-0.25	0.15	-0.26	37.51	-0.05	0.01	-0.12
HYAL3	-0.19	0.02	-0.19	0.22	-0.13	0.32	9.24	0.08	-0.01	0.39
HYAL4	-0.05	0.03	-0.56	-0.26	0.15	-2.42	0.01	-0.01	0.00	na
IDS	-0.27	0.07	-0.27	-0.26	0.15	-0.74	37.19	0.16	-0.02	0.37
IDUA	-0.24	0.01	-0.29	-0.13	0.07	-0.22	4.36	0.08	-0.01	0.60
MAN1A1	0.22	-0.01	0.18	-0.06	0.03	-0.14	13.93	-0.05	0.01	-0.28
MAN1A2	0.40	-0.04	0.23	0.49	-0.29	0.44	35.54	0.10	-0.01	0.25
MAN1B1	0.05	-0.01	0.03	-0.01	0.00	0.00	30.19	0.06	-0.01	0.16
MAN1C1	-0.17	0.07	-0.32	-0.11	0.06	-0.56	0.62	0.05	-0.01	0.95
MAN2A1	0.16	-0.01	0.11	0.31	-0.18	0.30	25.83	-0.07	0.01	-0.24

MAN2A2	-0.17	-0.03	-0.08	0.28	-0.16	0.25	12.43	0.06	-0.01	0.30
MAN2B1	0.20	-0.01	0.12	-0.02	0.01	-0.02	2.52	0.09	-0.01	0.90
MAN2B2	0.12	-0.01	0.06	0.00	0.00	0.00	12.67	0.19	-0.02	0.76
MAN2C1	-0.41	0.00	-0.29	-0.18	0.11	-0.18	15.97	0.09	-0.01	0.38
MANBA	0.64	-0.13	0.40	0.34	-0.20	0.41	13.86	0.19	-0.02	0.74
MGAT1	0.43	-0.03	0.22	-0.03	0.02	-0.02	39.01	0.27	-0.03	0.58
MGAT2	0.39	0.00	0.17	0.02	-0.01	0.01	24.36	0.13	-0.01	0.39
MGAT3	0.27	-0.02	0.39	-0.60	0.35	-2.00	1.70	-0.02	0.00	-0.35
MGAT4A	0.24	-0.03	0.16	0.19	-0.11	0.39	29.96	0.08	-0.01	0.21
MGAT4B	-0.64	0.10	-0.44	-0.39	0.23	-0.38	56.70	-0.29	0.03	-0.62
MGAT5	-0.21	-0.02	-0.20	-0.30	0.18	-0.65	29.65	-0.08	0.01	-0.26
MGAT5B	-0.11	0.02	-1.30	-0.20	0.12	-3.12	0.02	-0.01	0.00	na
MOGS	-0.30	0.01	-0.11	-0.01	0.01	-0.01	23.61	0.09	-0.01	0.28
NAGA	0.52	-0.04	0.23	0.40	-0.24	0.42	13.42	0.27	-0.03	0.99
NAGLU	-0.20	0.08	-0.18	-0.36	0.21	-0.34	12.23	0.04	0.00	0.18
NDST1	-0.05	0.01	-0.03	0.06	-0.03	0.08	6.64	-0.05	0.01	-0.36
NDST2	0.19	-0.03	0.09	0.42	-0.25	1.42	2.89	0.02	0.00	0.23
NDST3	-0.41	0.08	-0.92	-0.07	0.04	-0.73	0.01	-0.01	0.00	na
NDST4	-0.11	0.05	-1.87	-0.15	0.09	-5.97	0.02	-0.01	0.00	na
NEU1	-0.54	0.09	-0.60	-0.35	0.20	-0.60	52.08	0.20	-0.02	0.36
NEU2	-0.19	0.06	-1.53	-0.09	0.05	-0.46	0.01	-0.01	0.00	na
NEU3	-0.41	0.03	-0.33	0.09	-0.05	0.07	4.38	0.02	0.00	0.21
NEU4	-0.44	0.07	-1.32	0.03	-0.02	0.14	8.41	-0.06	0.01	-0.38
RFT1	-0.18	0.03	-0.08	0.29	-0.17	0.19	19.84	-0.07	0.01	-0.27
RPN1	0.60	-0.09	0.22	0.04	-0.03	0.02	67.19	0.41	-0.05	0.52
RPN2	-0.63	0.13	-0.42	-0.38	0.22	-0.37	77.64	-0.22	0.03	-0.27
SGSH	-0.21	0.02	-0.15	0.10	-0.06	0.15	6.35	0.09	-0.01	0.58
SLC33A1	0.27	0.00	0.11	0.12	-0.07	0.13	18.03	-0.01	0.00	-0.02
SPAM1	-0.14	0.04	-2.49	-0.14	0.08	-3.79	0.01	-0.01	0.00	na
ST3GAL1	-0.01	0.01	-0.03	-0.07	0.04	-0.30	7.31	-0.09	0.01	-0.82
ST3GAL2	-0.30	0.11	-0.26	-0.38	0.22	-0.67	10.16	-0.04	0.00	-0.27
ST3GAL3	-0.40	0.07	-0.40	-0.14	0.09	-0.31	1.47	0.02	0.00	0.27
ST3GAL4	0.26	-0.05	0.52	-0.04	0.03	-0.10	21.01	-0.07	0.01	-0.30
ST3GAL5	0.09	-0.01	0.13	-0.04	0.03	-0.16	1.17	0.18	-0.02	2.06
ST3GAL6	0.24	-0.01	0.32	-0.14	0.08	-1.70	0.26	0.05	-0.01	1.19
ST6GALNAC1	0.36	-0.06	0.61	0.28	-0.17	1.23	36.13	-0.21	0.02	-0.72
ST6GALNAC3	0.08	0.01	0.09	-0.14	0.08	-1.21	0.45	0.07	-0.01	1.46
ST6GALNAC4	0.25	-0.01	0.24	0.22	-0.13	0.67	18.12	0.32	-0.04	1.01
ST6GALNAC5	0.21	-0.02	0.37	-0.13	0.08	-0.91	0.03	-0.01	0.00	na
ST6GALNAC6	-0.06	0.01	-0.11	0.43	-0.25	0.90	6.78	0.04	0.00	0.24
ST8SIA1	-0.03	0.02	-0.09	0.14	-0.08	0.63	0.42	-0.03	0.00	-1.35
ST8SIA5	0.02	0.00	0.23	0.51	-0.30	2.64	0.05	0.07	-0.01	2.75
STT3A	0.68	-0.12	0.30	-0.03	0.02	-0.03	33.99	0.31	-0.04	0.68
STT3B	-0.22	0.01	-0.15	-0.21	0.12	-0.29	57.91	-0.06	0.01	-0.10
TUSC3	0.20	-0.03	0.38	-0.19	0.11	-1.92	1.00	-0.07	0.01	-3.06
UST	0.75	-0.10	1.44	0.40	-0.23	1.41	0.22	0.10	-0.01	2.42
XYLT1	-0.59	0.11	-0.60	-0.09	0.05	-0.25	5.35	-0.11	0.01	-1.19
XYLT2	-0.30	0.05	-0.20	-0.18	0.10	-0.19	6.59	0.03	0.00	0.21

Supplementary Table S4. Univariable and multivariable Cox regression analyses of *GALNT7* expression and clinicopathologic variables in the TCGA, AC-ICAM and GSE39582 cohorts, analyzed for all CRCs and separately for MSI and MSS CRCs

		Univariable			Multivariable		
		HR	95%CI	*P*	HR	95%CI	*P*
**TCGA all (n=566), OS**							
GALNT7	Low vs High	0.54	0.36 – 0.80	0.003	0.56	0.37 – 0.84	0.006
Age	Continuous	1.03	1.02 – 1.05	<0.001	1.04	1.03 – 1.06	<0.0001
Sex	Male vs Female	1.00	0.68 – 1.46	0.999	1.01	0.68 – 1.49	0.953
Tumor location	Colon vs Rectum	0.78	0.47 – 1.24	0.312	0.83	0.49 – 1.35	0.473
Histological type	AC vs MAC	1.26	0.69 – 2.13	0.426	1.36	0.72 – 2.37	0.313
Stage	I/II vs III/IV	3.72	2.46 – 5.79	<0.0001	4.44	2.89 – 7.01	<0.0001
**AC-ICAM all (n=348), PFS**							
GALNT7	Low vs High	0.53	0.35 – 0.78	0.002	0.64	0.42 – 0.97	0.037
Age	Continuous	0.99	0.97 – 1.01	0.173	1.00	0.98 – 1.01	0.558
Sex	Male vs Female	0.92	0.63 – 1.35	0.687	0.87	0.59 – 1.28	0.474
Tumor location	Right vs Left	0.80	0.54 – 1.17	0.256	0.67	0.45 – 0.99	0.047
Stage	I/II vs III/IV	5.48	3.49 – 8.99	<0.0001	5.09	3.21 – 8.41	<0.0001
**GSE39582 all (n=519), RFS**							
GALNT7	Low vs High	0.79	0.56 – 1.10	0.168	0.78	0.56 – 1.09	0.150
Age	Continuous	1.01	1.00 – 1.03	0.096	1.02	1.00 – 1.03	0.029
Sex	Male vs Female	0.82	0.58 – 1.15	0.256	0.75	0.53 – 1.06	0.101
Tumor location	Proxmimal vs Distal	1.16	0.82 – 1.64	0.408	1.25	0.88 – 1.80	0.214
Stage	I/II vs III/IV	2.32	1.65 – 3.28	<0.0001	2.39	1.70 – 3.39	<0.0001
**TCGA MSI (n=73), OS**							
GALNT7	Low vs High	0.23	0.04 – 0.93	0.065	0.24	0.04 – 1.01	0.079
Age	Continuous	1.06	1.00 – 1.14	0.092	1.05	1.00 – 1.13	0.120
Sex	Male vs Female	3.29	0.82 – 21.91	0.134	2.89	0.70 – 19.49	0.187
Tumor location	Colon vs Rectum		NA			NA	
Histological type	AC vs MAC		NA			NA	
Stage	I/II vs III/IV	1.44	0.31 – 5.20	0.600	1.71	0.36 – 6.43	0.451
**AC-ICAM MSI (n=57), PFS**							
GALNT7	Low vs High	0.14	0.02 – 0.54	0.012	0.20	0.03 – 0.84	0.047
Age	Continuous	1.01	0.99 – 1.04	0.371	0.99	0.95 – 1.04	0.698
Sex	Male vs Female	1.03	0.34 – 3.81	0.958	1.22	0.39 – 4.63	0.743
Tumor location	Right vs Left	1.28	0.52 – 2.70	0.554	0.83	0.18 – 2.86	0.788
Stage	I/II vs III/IV	8.69	2.65 – 38.78	0.001	6.53	1.93 – 29.71	0.005
**GSE39582 MSI (n=71), RFS**							
GALNT7	Low vs High	0.11	0.01 – 0.59	0.037	0.09	0.01 – 0.52	0.027
Age	Continuous	1.02	0.98 – 1.08	0.319	1.05	1.00 – 1.12	0.073
Sex	Male vs Female	0.21	0.032 – 0.84	0.049	0.15	0.02 – 0.69	0.028
Tumor location	Proxmimal vs Distal	0.36	0.020 – 1.92	0.333	0.80	0.04 – 6.40	0.855
Stage	I/II vs III/IV	1.16	0.30 – 4.05	0.823	1.06	0.27 – 3.77	0.928
**TCGA MSS (n=430), OS**							
GALNT7	Low vs High	0.58	0.37 to 0.90	0.017	0.63	0.39 to 1.00	0.054
Age	Continuous	1.04	1.02 to 1.06	<0.0001	1.05	1.03 to 1.07	<0.0001
Sex	Male vs Female	0.97	0.63 to 1.50	0.898	0.89	0.57 to 1.40	0.625
Tumor location	Colon vs Rectum	0.82	0.49 to 1.34	0.453	0.99	0.56 to 1.66	0.958
Histological type	AC vs MAC	1.94	0.97 to 3.51	0.041	1.97	0.96 to 3.70	0.046
Stage	I/II vs III/IV	3.54	2.19 to 5.92	<0.0001	4.19	2.54 to 7.20	<0.0001
**AC-ICAM MSS (n=224), PFS**							
GALNT7	Low vs High	0.56	0.34 to 0.90	0.018	0.68	0.41 to 1.13	0.143
Age	Continuous	0.99	0.97 to 1.01	0.248	1.00	0.98 to 1.02	0.922
Sex	Male vs Female	0.98	0.61 to 1.56	0.927	0.99	0.61 to 1.60	0.973
Tumor location	Right vs Left	0.79	0.49 to 1.26	0.317	0.73	0.45 to 1.19	0.206
Stage	I/II vs III/IV	4.12	2.44 to 7.32	<0.0001	3.76	2.18 to 6.81	<0.0001
**GSE39582 MSS (n=405), RFS**							
GALNT7	Low vs High	0.87	0.61 to 1.24	0.430	0.90	0.632 to 1.29	0.579
Age	Continuous	1.01	1.00 to 1.03	0.144	1.02	1.00 to 1.03	0.050
Sex	Male vs Female	1.00	0.69 to 1.42	0.984	0.91	0.63 to 1.31	0.620
Tumor location	Proxmimal vs Distal	0.92	0.63 to 1.33	0.671	0.85	0.57 to 1.23	0.395
Stage	I/II vs III/IV	2.23	1.55 to 3.26	<0.0001	2.31	1.60 to 3.39	<0.0001

HR, hazard ratio; CI, confidence interval; AC. Adenocarcinoma; MAC, mucinous adenocarcinoma; NA, not applicable

Supplementary Table S5. Correlation between GALNT7 expression and Immune ESTIMATE, and immune checkpoint genes in MSI CRC using three independent datasets

	**TCGA MSI (n=76)**			**AC-ICAM MSI (n=57)**			**GSE39582 MSI (n=75)**		
	r	95% CI	*P*	*r*	95% CI	*P*	*r*	95% CI	*P*
GALNT7 vs. Immune ESTIMATE	-0.3699	-0.5495 to -0.1576	**0.0010**	-0.4097	-0.6056 to -0.1669	**0.0016**	-0.2431	-0.4455 to -0.01705	**0.0356**
GALNT7 vs. *CTLA4* (CTLA-4)	-0.3346	-0.5208 to -0.1180	**0.0031**	-0.2355	-0.4674 to 0.02670	0.0778	-0.4366	-0.6038 to -0.2327	**<0.0001**
GALNT7 vs. *HAVCR2* (TIM-3)	-0.2839	-0.4787 to -0.06241	**0.0130**	-0.4429	-0.6307 to -0.2061	**0.0006**	-0.4164	-0.5878 to -0.2092	**0.0002**
GALNT7 vs. *CD274* (PD-L1)	-0.3094	-0.5000 to -0.09022	**0.0065**	-0.4776	-0.6565 to -0.2479	**0.0002**	-0.2665	-0.4653 to -0.04211	**0.0208**
GALNT7 vs. *PDCD1LG2* (PD-L2)	-0.1371	-0.3517 to 0.09116	0.2376	-0.4885	-0.6645 to -0.2612	**0.0001**	-0.1349	-0.3511 to 0.09496	0.2485
GALNT7 vs. *LAG3* (LAG-3)	-0.3674	-0.5475 to -0.1548	**0.0011**	-0.3242	-0.5392 to -0.06947	**0.0139**	-0.4586	-0.6210 to -0.2586	**<0.0001**
GALNT7 vs. *PDCD1* (PD-1)	-0.3872	-0.5635 to -0.1772	**0.0005**	-0.4110	-0.6066 to -0.1685	**0.0015**	-0.2334	-0.4372 to -0.006775	**0.0439**

Supplementary Table S6. Clinicopathological characteristics of patients with CRC according to GALNT7 expression by IHC

	Total n=619	GALNT7-Low n=464 (75.0%)	GALNT7-High n=155 (25.0%)	*p*
Age				0.6452
Mean±SD	68.4±11.7	68.3±11.4	68.8±12.5	
Sex				0.0554
Male	386 (62.4%)	279 (60.1%)	107 (69.0%)	
Female	233 (37.6%)	185 (39.9%)	48 (31.0%)	
Location				0.2314
Proximal colon	233 (37.6%)	165 (35.6%)	68 (43.9%)	
Distal colon	169 (27.3%)	135 (29.1%)	34 (21.9%)	
Rectum	217 (25.1%)	164 (35.3%)	53 (34.2%)	
Tumor differentiation				0.5352
Well-Moderately	586 (94.7%)	441 (95.0%)	145 (93.5%)	
Poorly	33 (5.3%)	23 (5.0%)	10 (6.5%)	
Histology				0.0565
Non-mucinous	587 (94.8%)	445 (95.9%)	142 (91.6%)	
Mucinous	32 (5.2%)	19 (4.1%)	13 (8.4%)	
Tumor invasion				0.9955
Tis	32 (5.2%)	20 (4.3%)	12 (7.7%)	
T1	82 (13.2%)	63 (13.6%)	19 (12.3%)	
T2	91 (14.7%)	72 (15.5%)	19 (12.3%)	
T3	239 (38.6%)	183 (39.4%)	56 (36.1%)	
T4	175 (28.3%)	126 (27.2%)	49 (31.6%)	
Lymphatic invasion				0.0301
Absent	213 (34.4%)	149 (32.1%)	64 (41.3%)	
Present	399 (64.5%)	312 (67.2%)	87 (56.1%)	
Not available	7 (1.1%)	3 (0.6%)	4 (2.6%)	
Venous invasion				0.3906
Absent	157 (25.4%)	114 (24.6%)	43 (27.7%)	
Present	455 (73.5%)	347 (74.8%)	108 (69.7%)	
Not available	7 (1.1%)	3 (0.6%)	4 (2.6%)	
Lymph node metastasis				0.1239
Absent	388 (62.7%)	282 (60.8%)	106 (68.4%)	
Present	228 (36.8%)	179 (38.6%)	49 (31.6%)	
Not available	3 (0.5%)	3 (0.6%)	0 (0.0%)	
Distant metastasis				0.0763
Absent	535 (86.4%)	394 (84.9%)	141 (91.0%)	
Present	83 (12.4%)	69 (14.9%)	14 (9.0%)	
Not available	1 (0.2%)	1 (0.2%)	0 (0.0%)	
Stage				0.0509
Stage 0	31 (5.0%)	20 (4.3%)	11 (7.1%)	
Stage I	144 (23.3%)	111 (23.9%)	33 (21.3%)	
Stage II	189 (30.5%)	130 (28.0%)	59 (38.1%)	
Stage III	170 (27.5%)	131 (28.2%)	39 (25.2%)	
Stage IV	84 (13.6%)	71 (15.3%)	13 (8.4%)	
Not available	1 (0.2%)	1 (0.2%)	0 (0.0%)	

Supplementary Table S7. Clinicopathological characteristics of patients with dMMR/MSI CRC according to GALNT7 expression by IHC

	Total n=52	dMMR/MSI, GALNT7-Low n=18 (34.6%)	dMMR/MSI, GALNT7-High n=34 (65.4%)	*p*
Age				0.2909
Mean±SD	67.3±15.0	64.3±13.5	68.8±15.7	
Sex				0.1441
Male	26 (50.0%)	6 (33.3%)	20 (58.8%)	
Female	26 (50.0%)	12 (66.7%)	14 (41.2%)	
Location				0.0784
Proximal colon	42 (80.8%)	11 (61.1%)	31 (91.2%)	
Distal colon	5 (9.6%)	5 (27.8%)	0 (0.0%)	
Rectum	5 (9.6%)	2 (11.1%)	3 (8.8%)	
Tumor differentiation				0.0402
Well-Moderately	39 (75.0%)	10 (55.6%)	29 (85.3%)	
Poorly	13 (25.0%)	8 (44.4%)	5 (14.7%)	
Histology				>0.9999
Non-mucinous	47 (90.4%)	16 (88.9%)	31 (91.2%)	
Mucinous	5 (9.6%)	2 (11.1%)	3 (8.8%)	
Tumor invasion				0.7951
T1	5 (9.6%)	1 (5.6%)	4 (11.8%)	
T2	9 (17.3%)	4 (22.2%)	5 (14.7%)	
T3	27 (51.9%)	9 (50.0%)	18 (52.9%)	
T4	11 (21.2%)	4 (22.2%)	7 (20.6%)	
Lymphatic invasion				0.0365
Absent	22 (42.3%)	4 (22.2%)	18 (52.9%)	
Present	28 (53.8%)	14 (77.8%)	14 (41.2%)	
Not available	2 (3.8%)	0 (0.0%)	2 (5.9%)	
Venous invasion				0.1990
Absent	15 (28.8%)	3 (16.7%)	12 (35.3%)	
Present	35 (67.3%)	15 (83.3%)	20 (58.8%)	
Not available	2 (3.8%)	0 (0.0%)	2 (5.9%)	
Lymph node metastasis				0.0524
Absent	38 (73.1%)	10 (55.6%)	28 (82.4%)	
Present	14 (26.9%)	8 (44.4%)	6 (17.6%)	
Distant metastasis				>0.9999
Absent	50 (96.2%)	17 (94.4%)	33 (97.1%)	
Present	2 (3.8%)	1 (5.6%)	1 (2.9%)	
Stage				0.1461
Stage I	13 (25.0%)	4 (22.2%)	9 (26.5%)	
Stage II	25 (48.1%)	6 (33.3%)	19 (55.9%)	
Stage III	12 (23.1%)	7 (38.9%)	5 (14.7%)	
Stage IV	2 (3.8%)	1 (5.6%)	1 (2.9%)	

Supplementary Figure S1

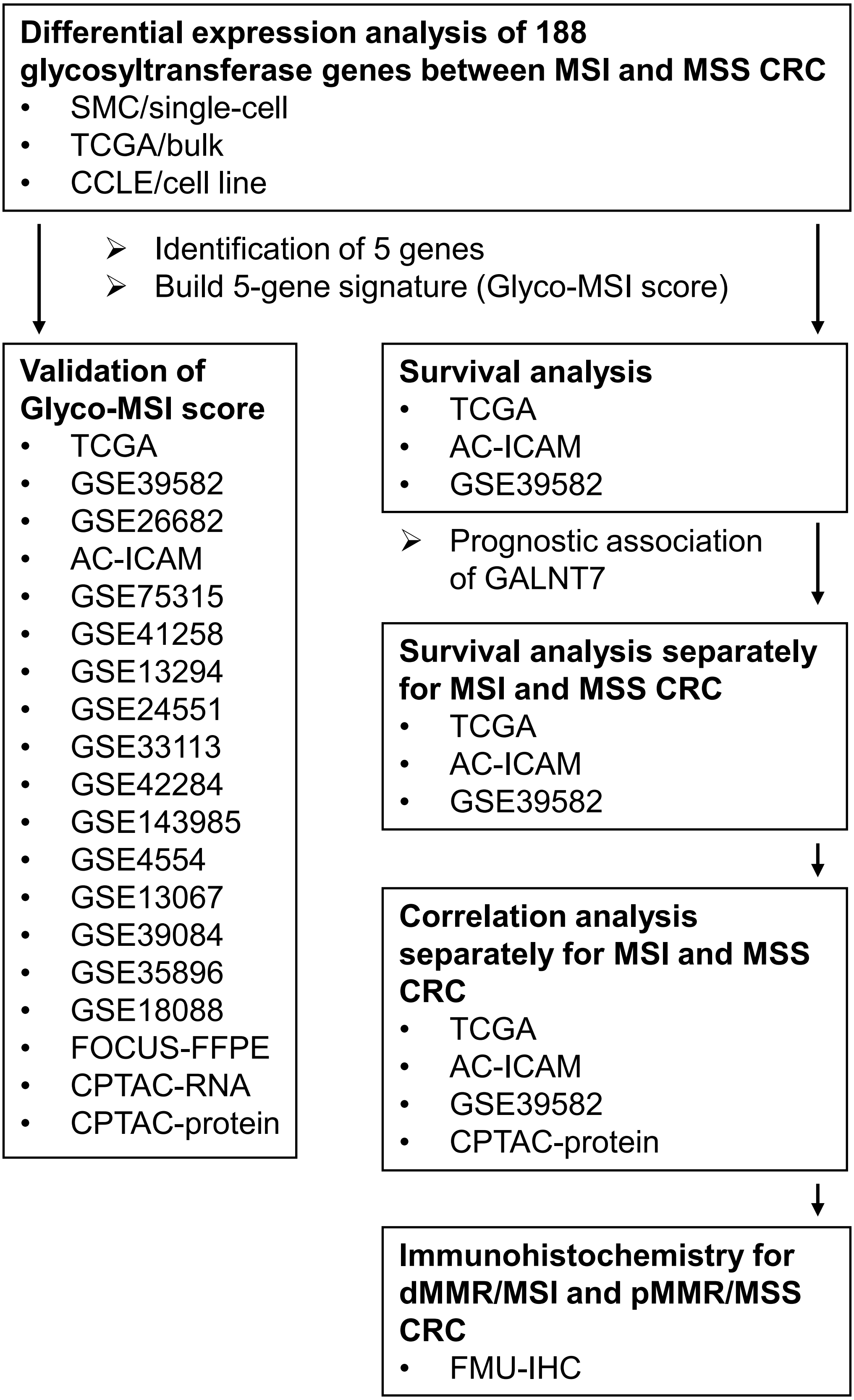

Supplementary Figure S1. Study flowchart.

Supplementary Figure S2

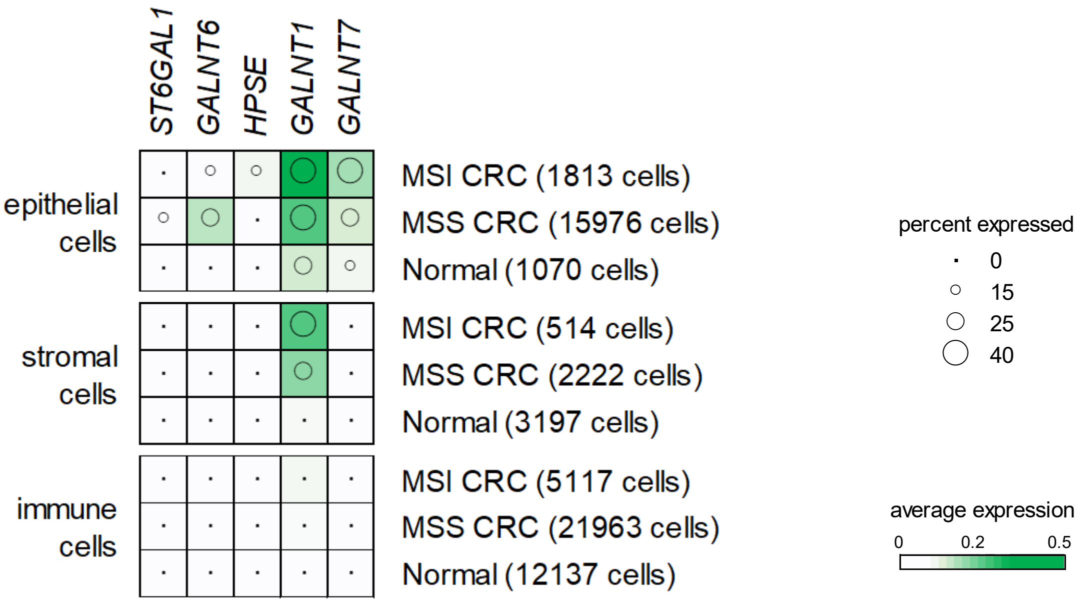

Supplementary Figure S2. Heatmap represents the average expression levels of five glycosyltransferase genes and dot size corresponds to the percentage of cells expressing the gene in each cell type, including epithelial, stomal and immune single-cells from MSI CRC, MSS CRC or normal mucosa, based on the SMC single-cell RNA-seq data.

Supplementary Figure S3

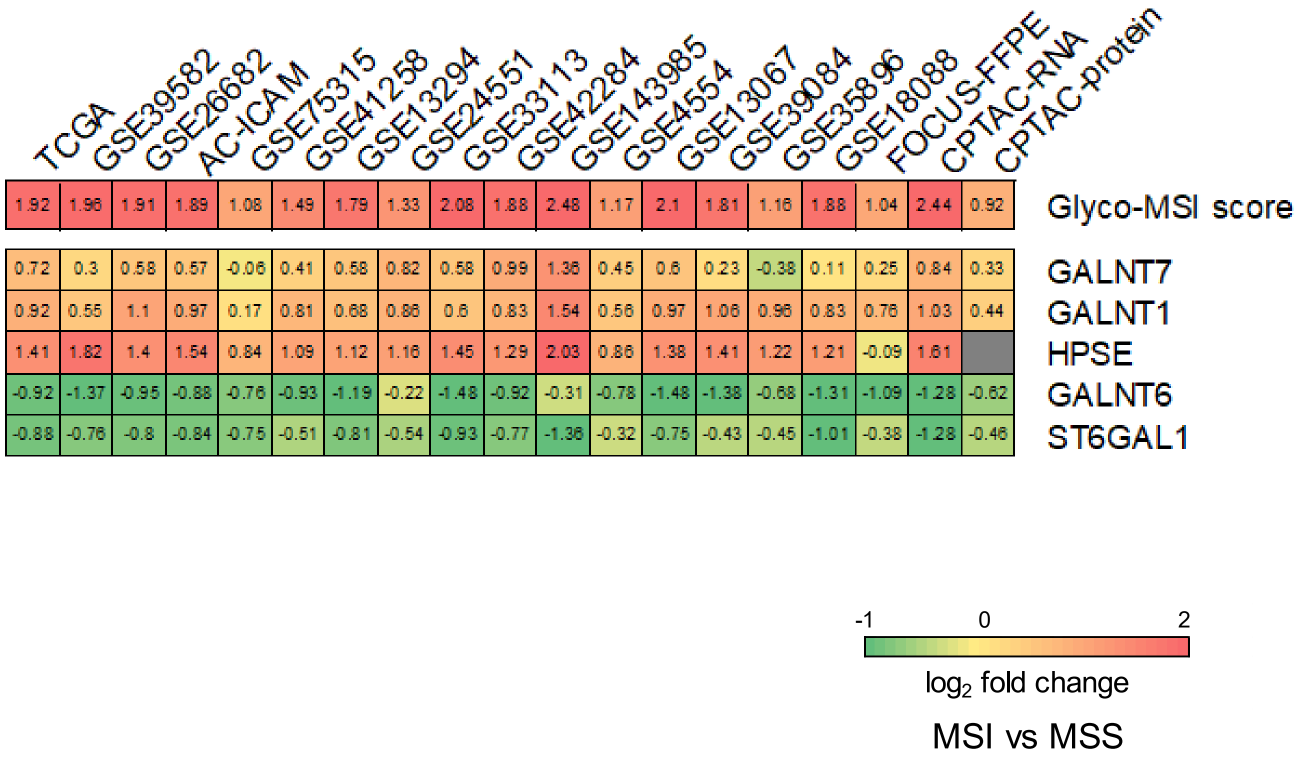

Supplementary Figure S3. Heatmap illustrating the differential expression (log_2 fold change) of the Glyco-MSI score and the five genes between MSI and MSS CRCs in 19 transcriptomic (RNA-seq or microarray) and proteomic (CPTAC-protein by mass-spectrometry) cohorts. This analysis contained a total of 587 MSI and 2862 MSS samples of bulk CRC.

Supplementary Figure S4

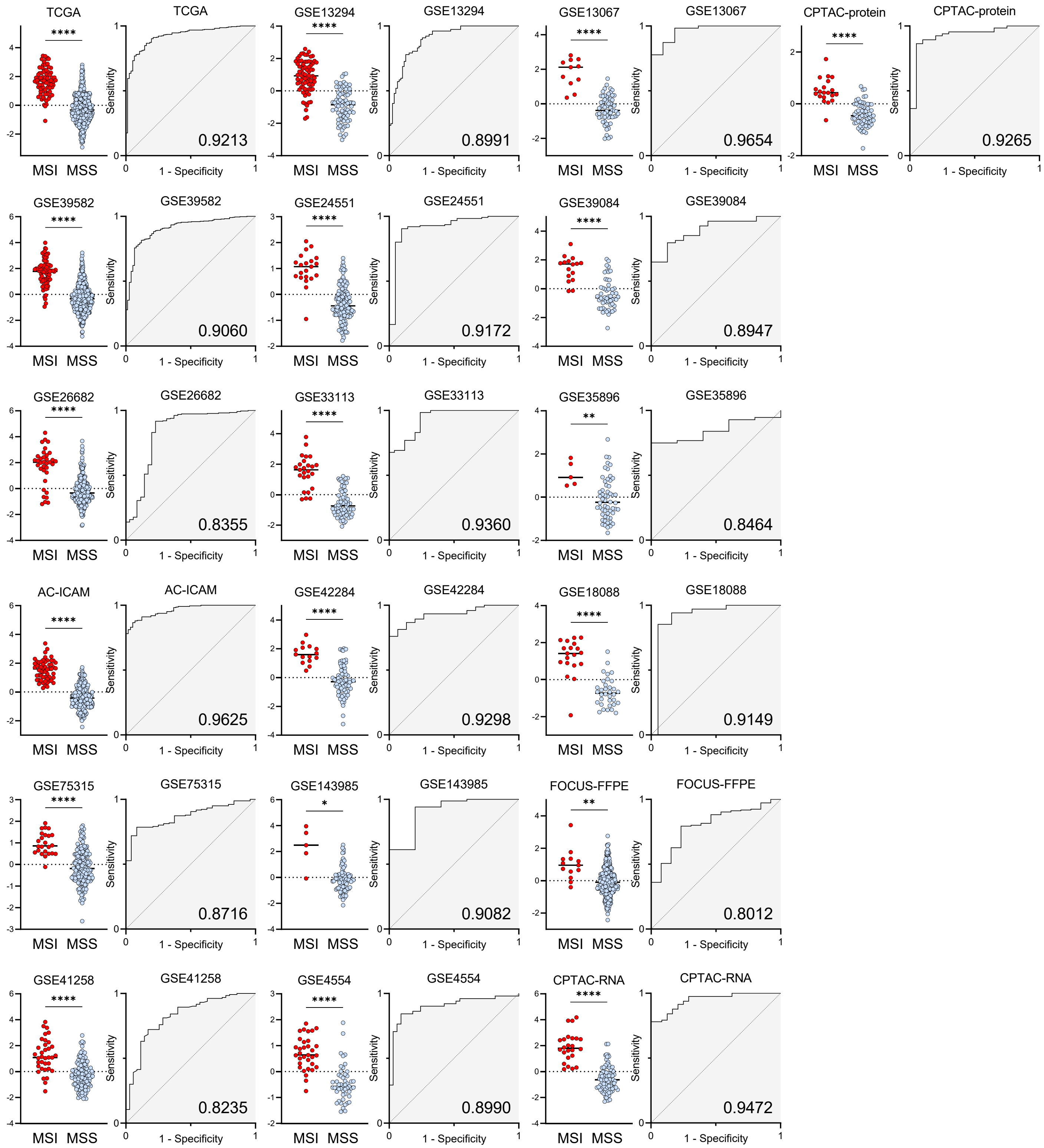

Supplementary Figure S4. Levels of the Glyco-MSI score in MSI and MSS CRCs (Left) and ROC curves (Right) to assess the performance of the Glyco-MSI score in 19 independent cohorts. ****$P < 0.0001$, ***$P < 0.001$, **$P < 0.01$, *$P < 0.05$. AUC values are presented for each cohort.

Supplementary Figure S5

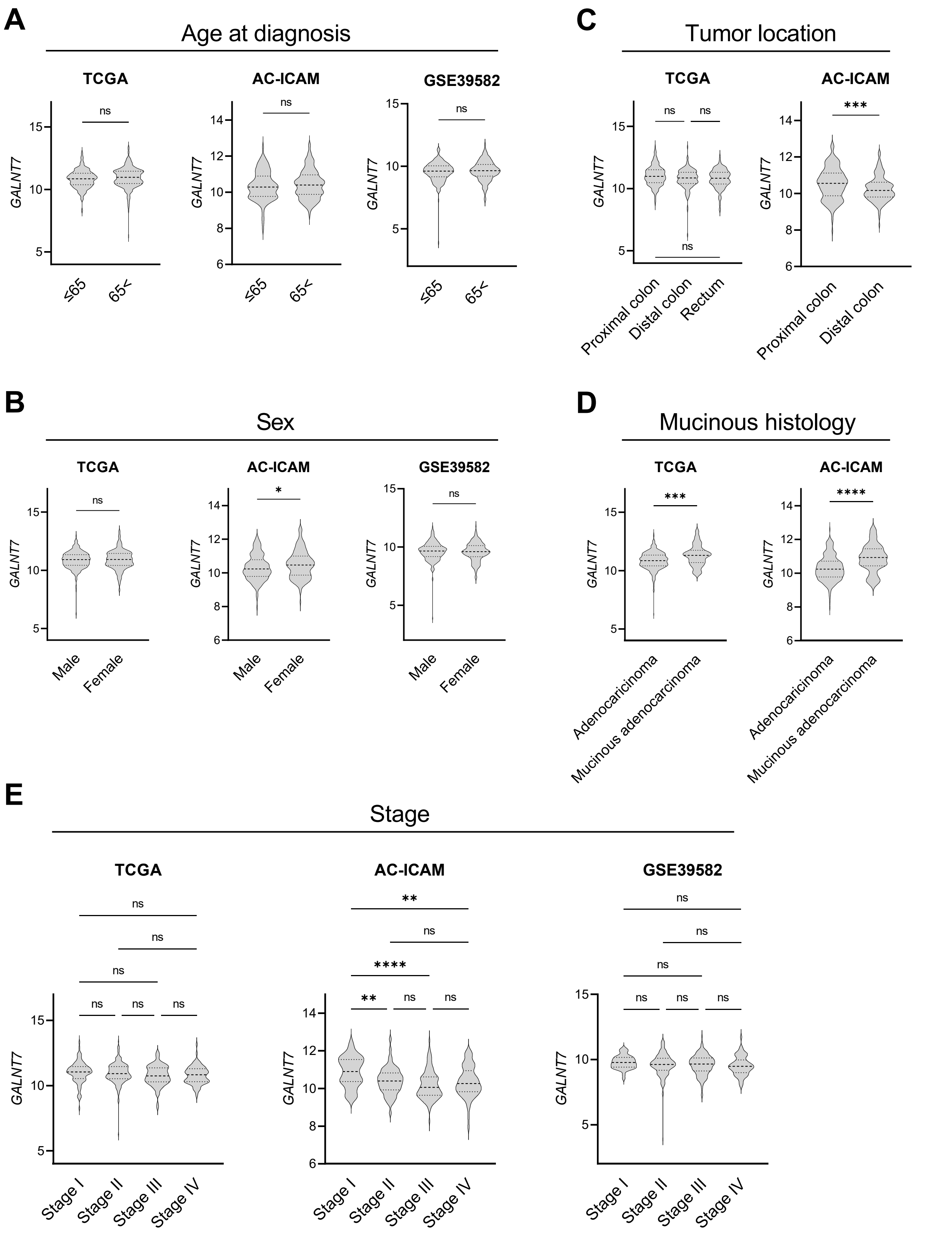

Supplementary Figure S5. Association of *GALNT7* expression with age at diagnosis (A), sex (B), tumor location (C), mucinous histology (D), and stage of disease (E) in TCGA, AC-ICAM and GSE39582. ****$P < 0.0001$, ***$P < 0.001$, **$P < 0.01$, *$P < 0.05$, n.s. $P > 0.05$.

Supplementary Figure S6

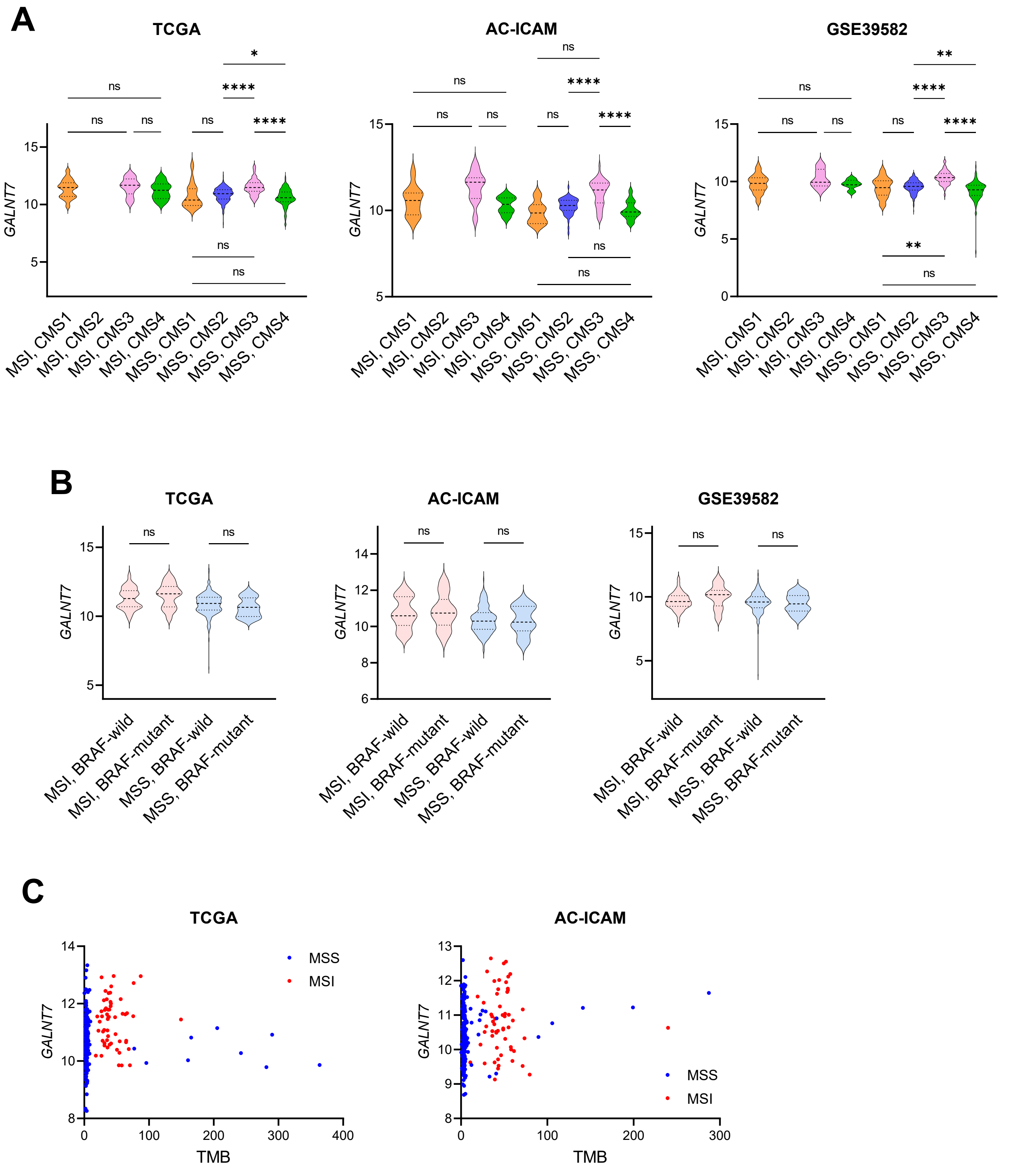

Supplementary Figure S6. Association of *GALNT7* expression with consensus molecular subtypes (CMSs) (A), *BRAF* mutations (B) and tumor mutation burden (TMB) (C), stratified by MSI status, in TCGA, AC-ICAM and GSE39582. ****$P < 0.0001$, ***$P < 0.001$, **$P < 0.01$, *$P < 0.05$, n.s. $P > 0.05$.

Supplementary Figure S7

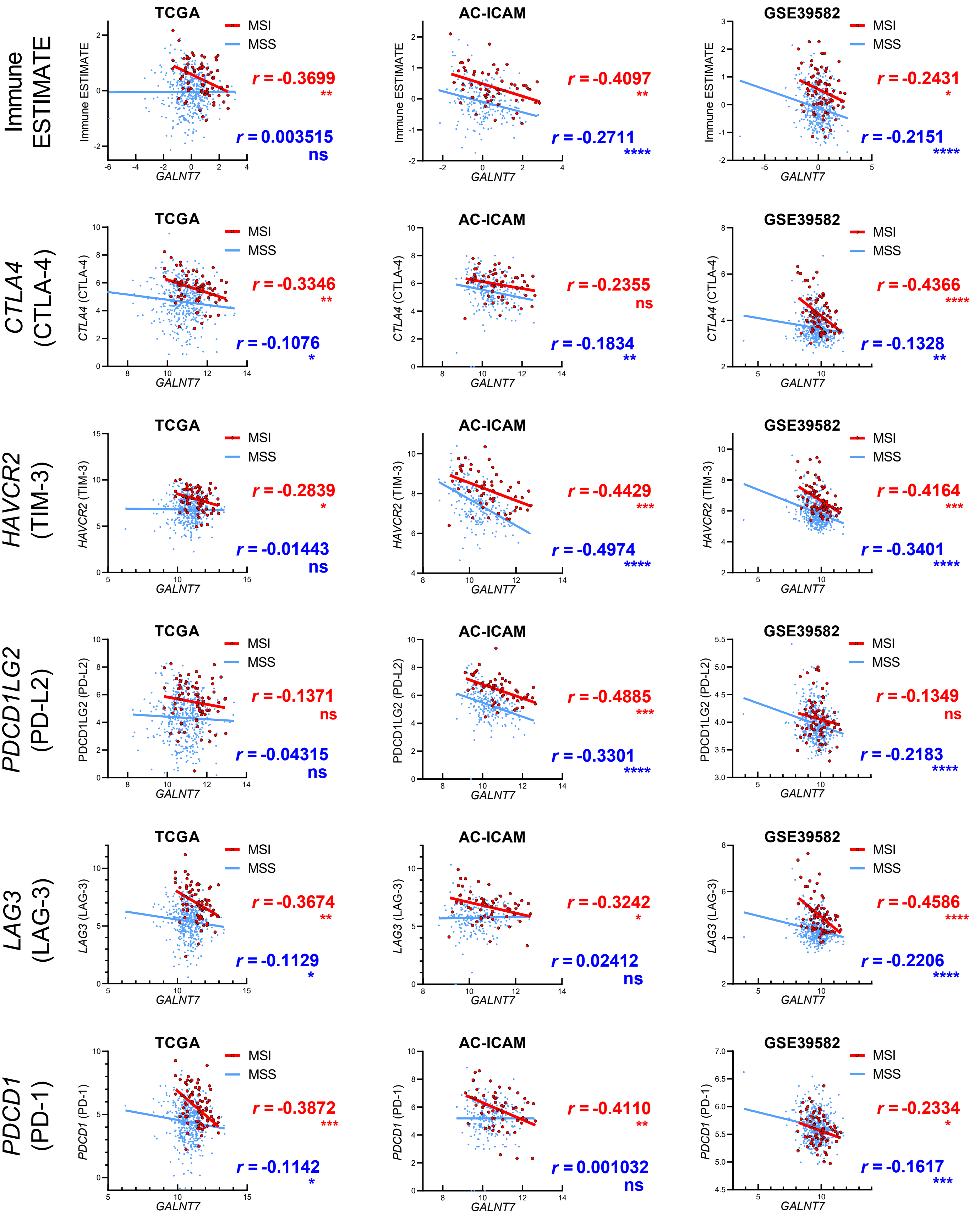

Supplementary Figure S7. Correlations of *GALNT7* expression with the levels of immune infiltration (ESTIMATE based on 141 immune genes) and immune checkpoint genes, stratified by MSI status, in TCGA, AC-ICAM and GSE39582. Pearson correlation coefficients (*r*) are indicated. ****$P < 0.0001$, ***$P < 0.001$, **$P < 0.01$, *$P < 0.05$, n.s. $P > 0.05$.

Supplementary Figure S8

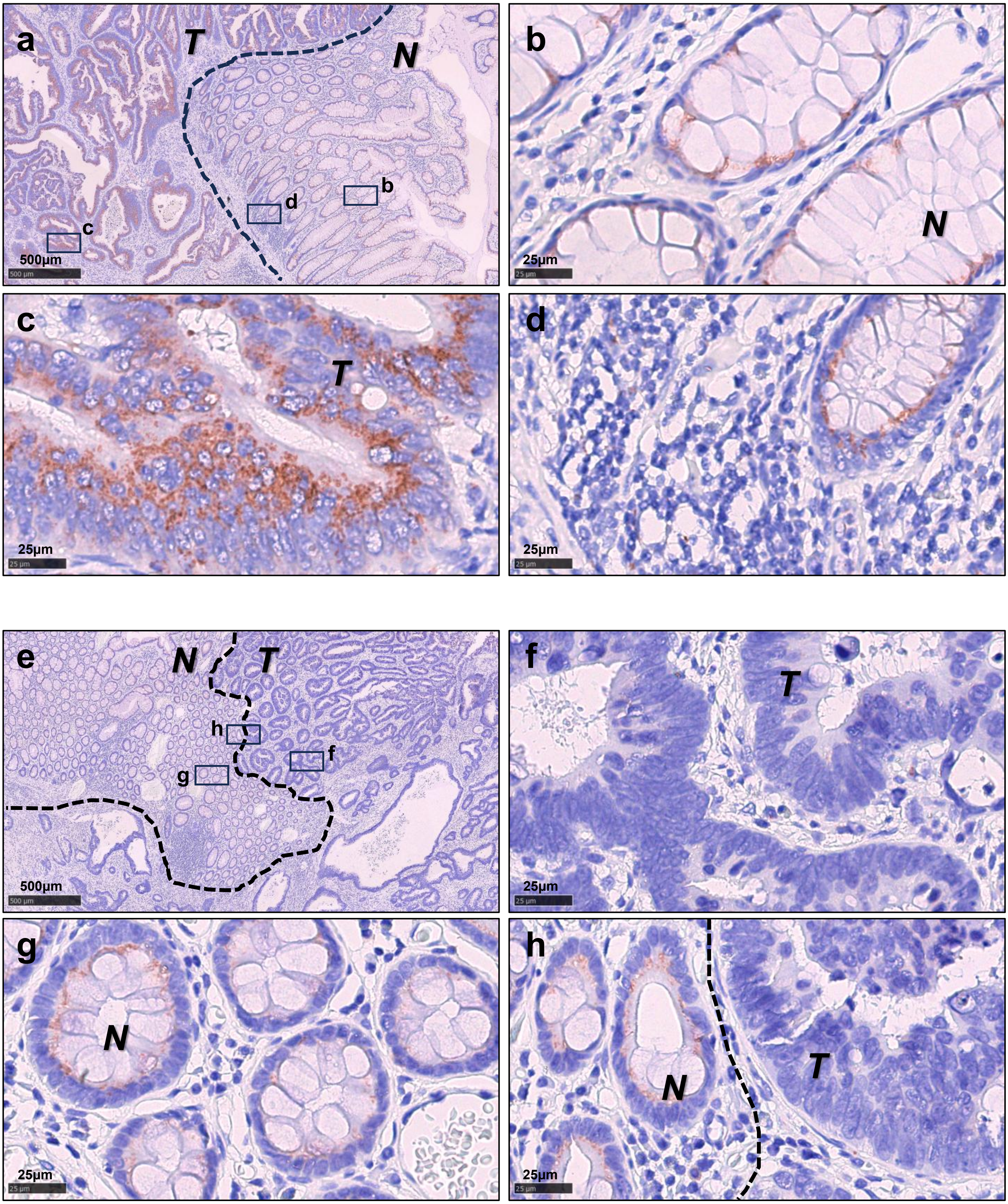

Supplementary Figure S8. IHC for GALNT7 in CRC tissues [T] and adjacent normal tissues [N]. **A-D,** A representative case of CRC (A), showing relatively weak cytoplasmic staining in normal epithelial cells (B), strong cytoplasmic staining in tumor cells (C), and no staining in stromal cell or immune cells (D). **E-F,** A representative case of CRC (E), showing weak staining in normal epithelial cells (G, H), but lacking GALNT7 staining in tumor cells (F, H).

Supplementary Figure S9

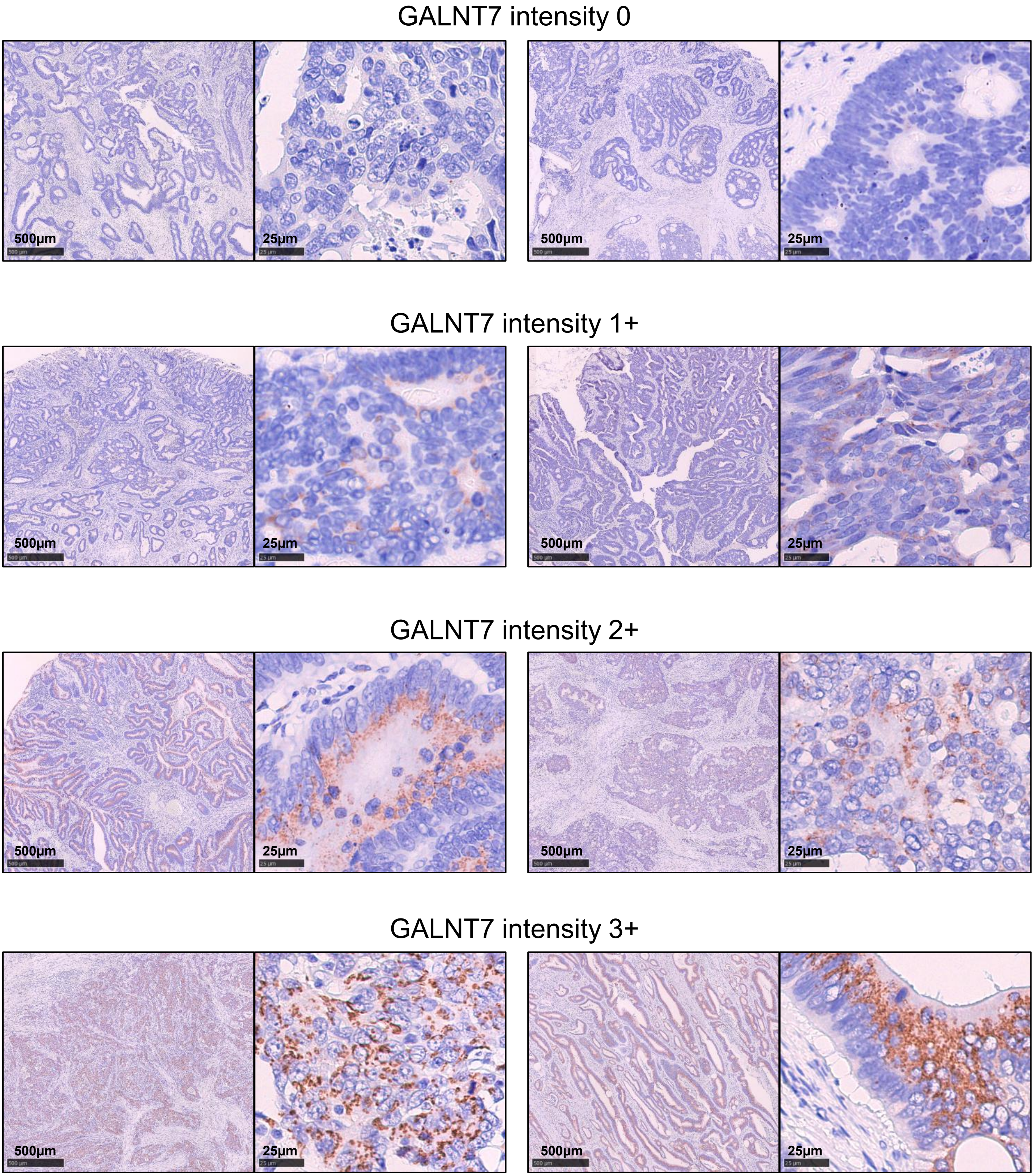

Supplementary Figure S9. Representative intensities of GALNT7 staining in CRC.

Supplementary Figure S10

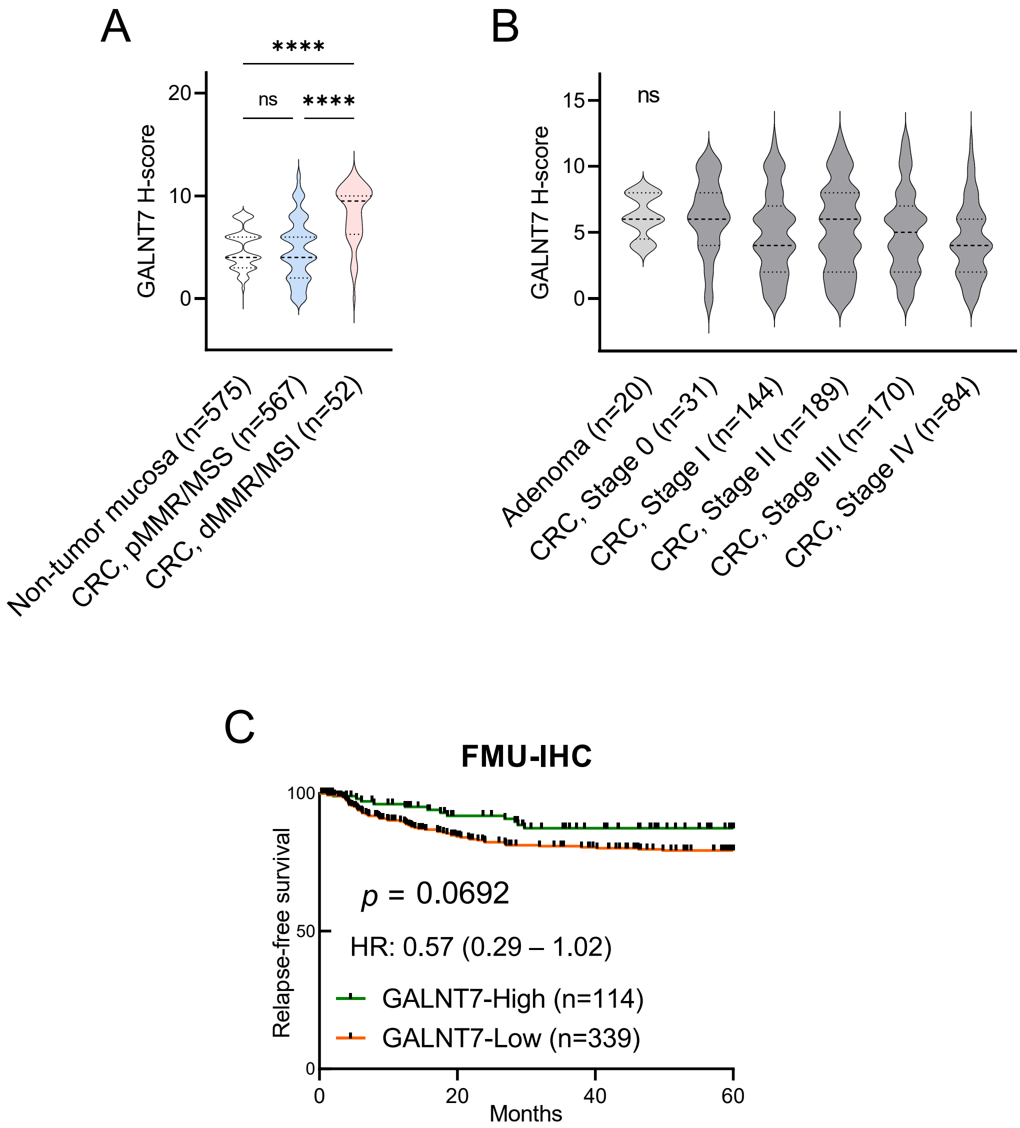

Supplementary Figure S10. Associations of GALNT7 expression by IHC with MSI/MMR status, disease stage and prognosis. **A,** GALNT7 H-score in non-tumor mucosa, pMMR/MSS CRC and dMMR/MSI CRC. **B,** GALNT7 H-score in adenomas and CRCs with different stages of disease. ****$P < 0.0001$, n.s. $P > 0.05$. **C,** Kaplan-Meier curves for RFS according to GALNT7 IHC in FFPE cohort (FMU-IHC). Log-rank *p* value, Cox hazard ratio (HR) and 95% confidence interval are indicated.

Supplementary Figure S11

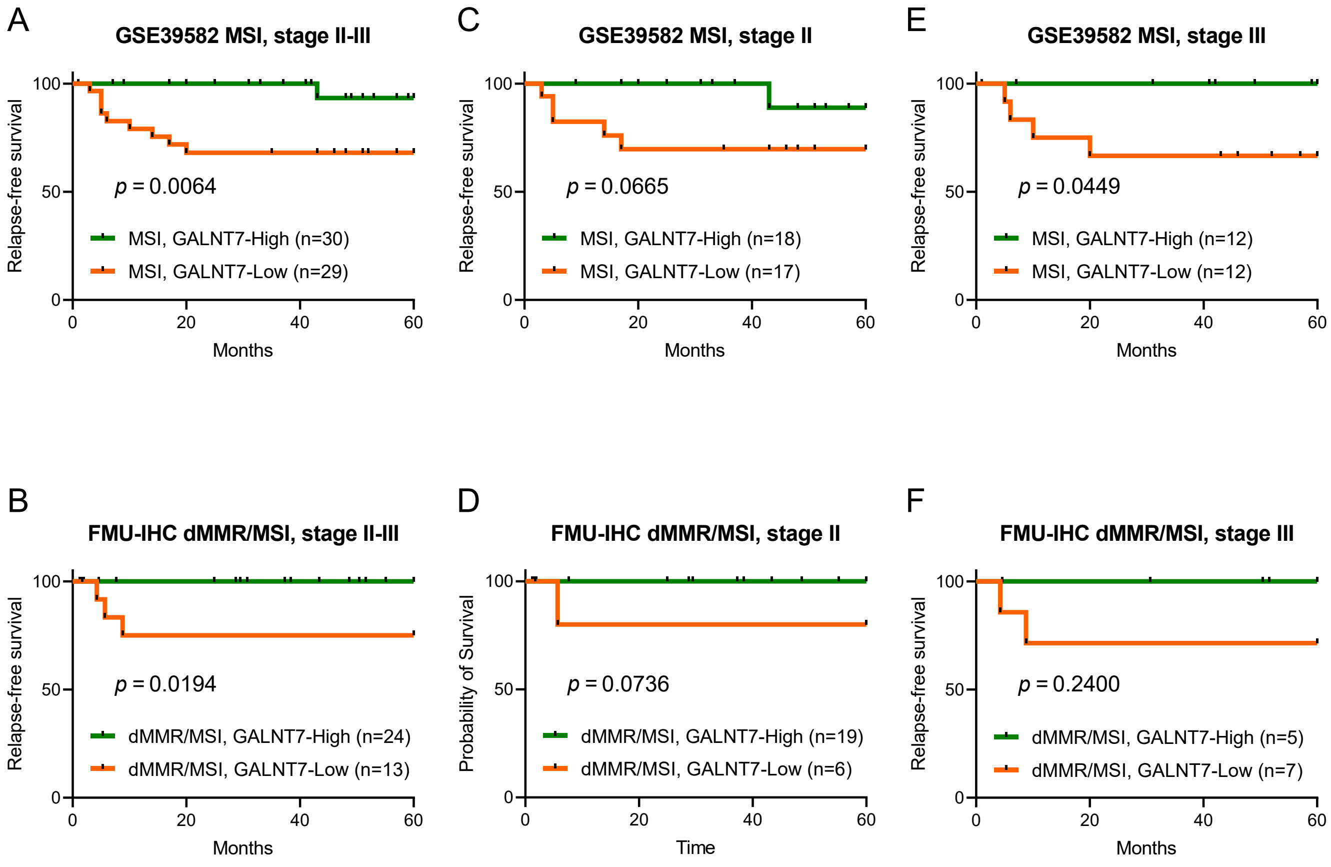

Supplementary Figure S11. RFS analyses for patients with stage II-III dMMR/MSI CRC. Kaplan-Meier curves for RFS according to GALNT7 expression in stage II-III (**A, B**), and stratified stage II (**C, D**) and stage III (**E, F**) in the GSE39582 MSI cohort (**A, C, E**) and the FMU-IHC dMMR/MSI cohort (**B, D, F**). Log-rank *p* values are indicated.

Supplementary Figure S12

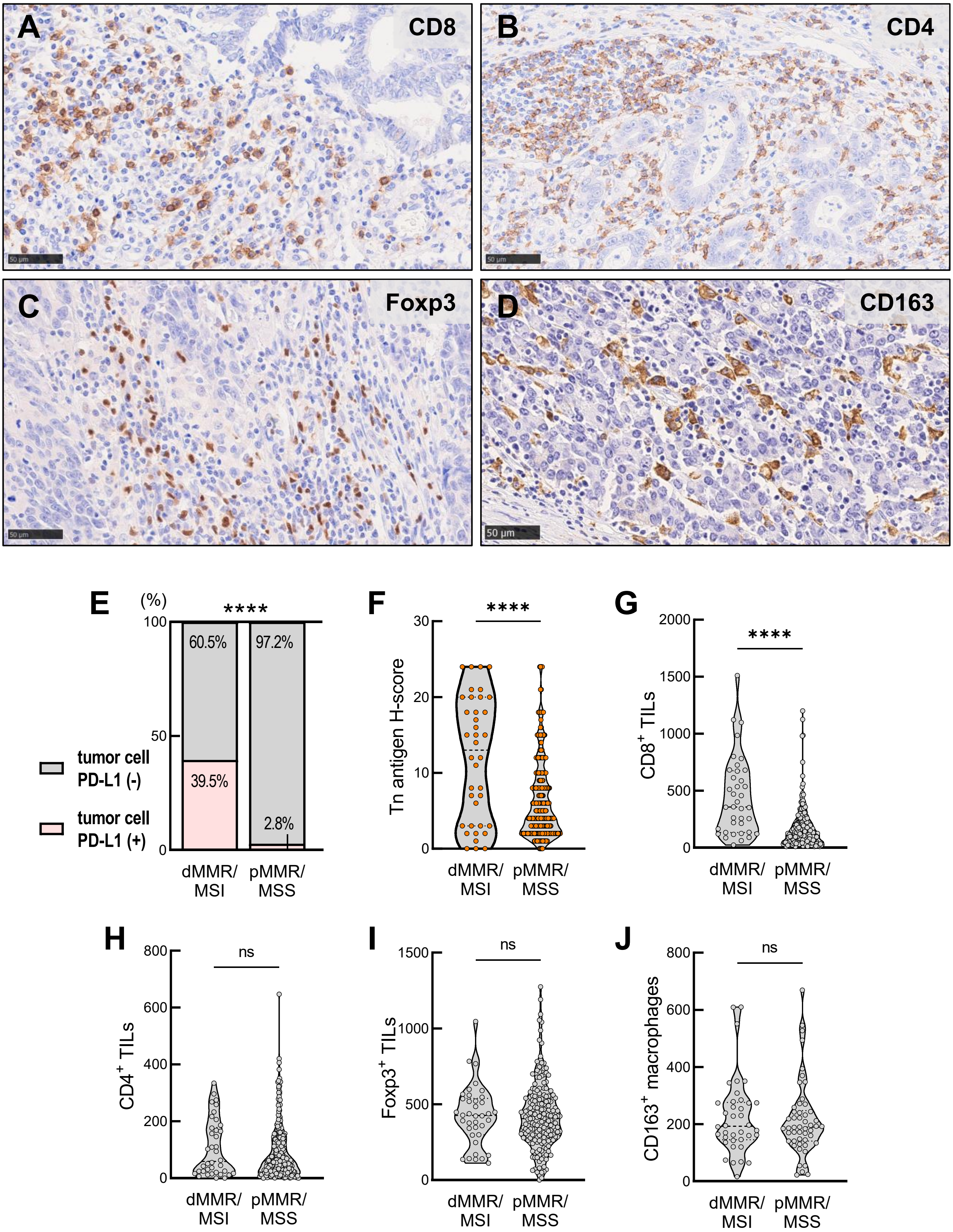

Supplementary Figure S12. Immune infiltration, PD-L1 expression and Tn antigen in CRC by IHC. **A-D,** Representative IHC images of CD8 (A), CD4 (B), Foxp3 (C) and CD163 (D). **E-J,** Relative proportion of tumor cell PD-L1 positivity (E), Tn antigen H-score (F), $CD8^+$ TILs (G), $CD4^+$ TILs (H), $Foxp3^+$ TILs (I) and $CD163^+$ macrophages (J) in dMMR/MSI and pMMR/MSS CRC. ****$P < 0.0001$, n.s. $P > 0.05$.

Supplementary Figure S13

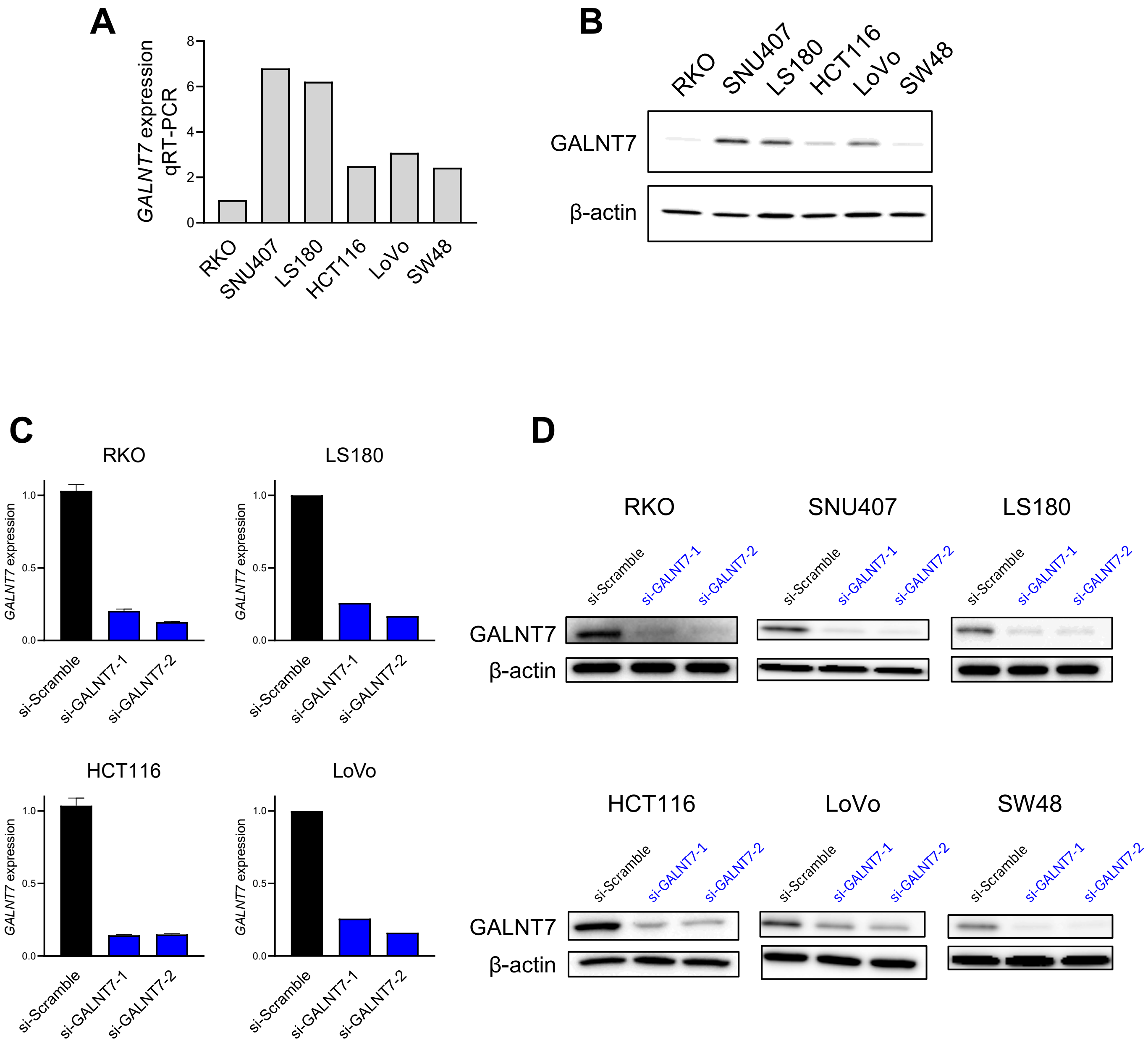

Supplementary Figure S13. A,B, GALNT7 mRNA and protein levels in six MSI CRC cell lines determined by qRT-PCR (A) and western blotting analysis (B). **C,D,** Cell lines were transfected with siRNAs targeting GALNT7 or scramble negative control, showing effectively decreased expression of GALNT7, confirmed by qRT-PCR (C) and western blotting (D).

Supplementary Figure S14

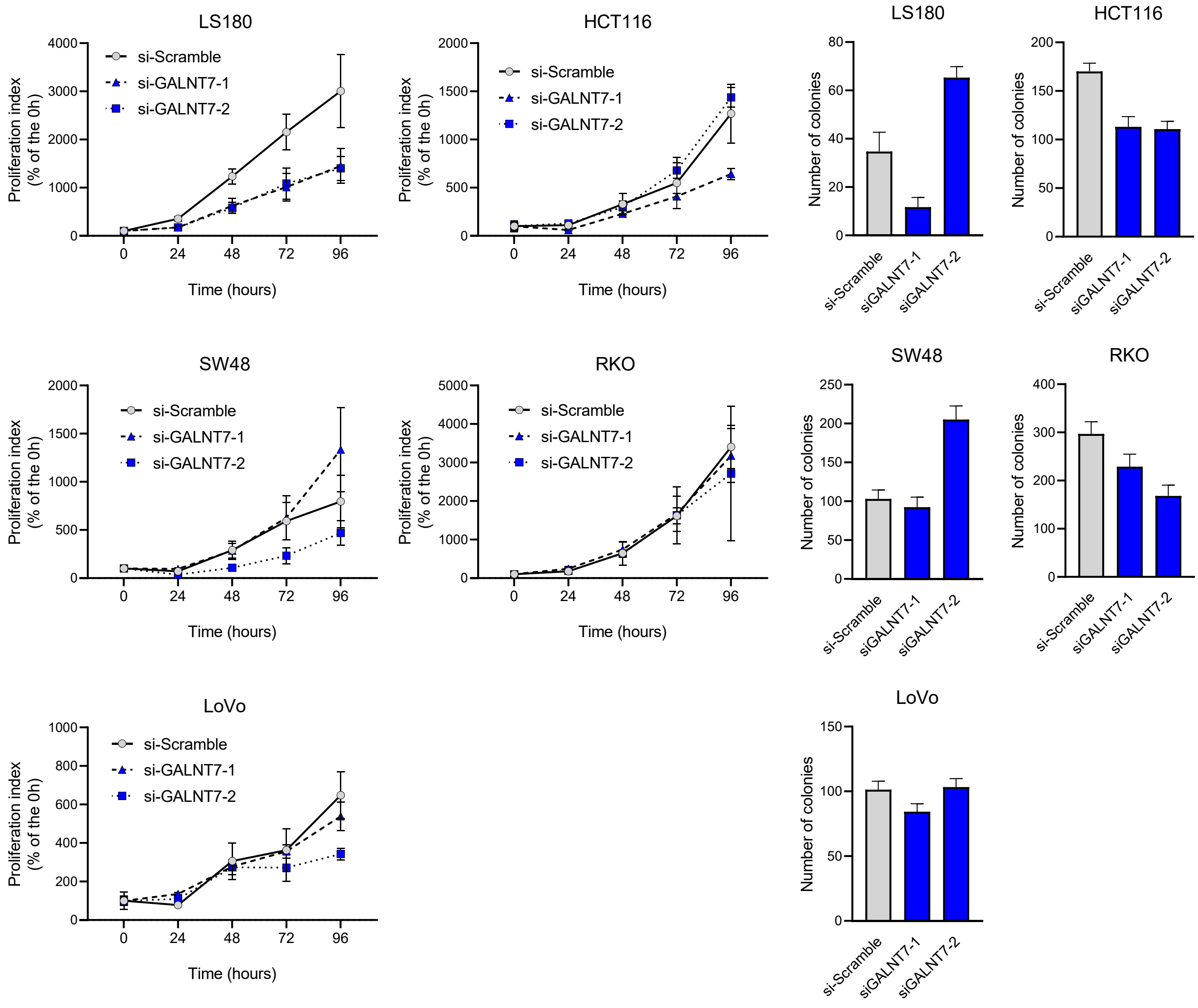

Supplementary Figure S14. Cell proliferation and colony formation assays for CRC cell lines, transfected with siRNAs targeting GALNT7 or scramble negative control.

Supplementary Figure S15

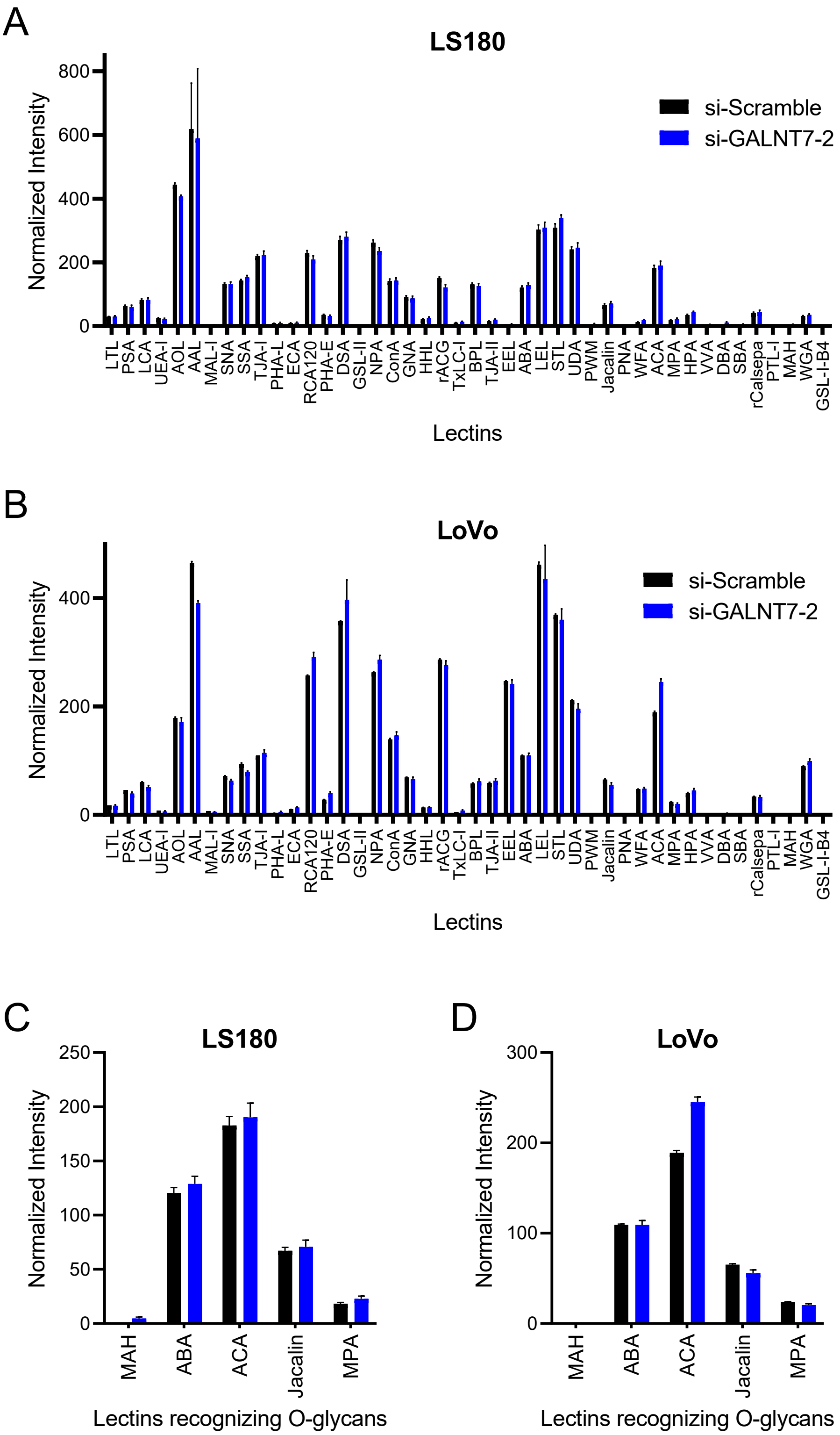

Supplementary Figure S15. Cell surface glycan profile were analyzed by lectin microarray using LS180 (A,C) and LoVo (B,D) cell lines transfected with siRNAs targeting scramble negative control or GALNT7.

Supplementary Figure S16

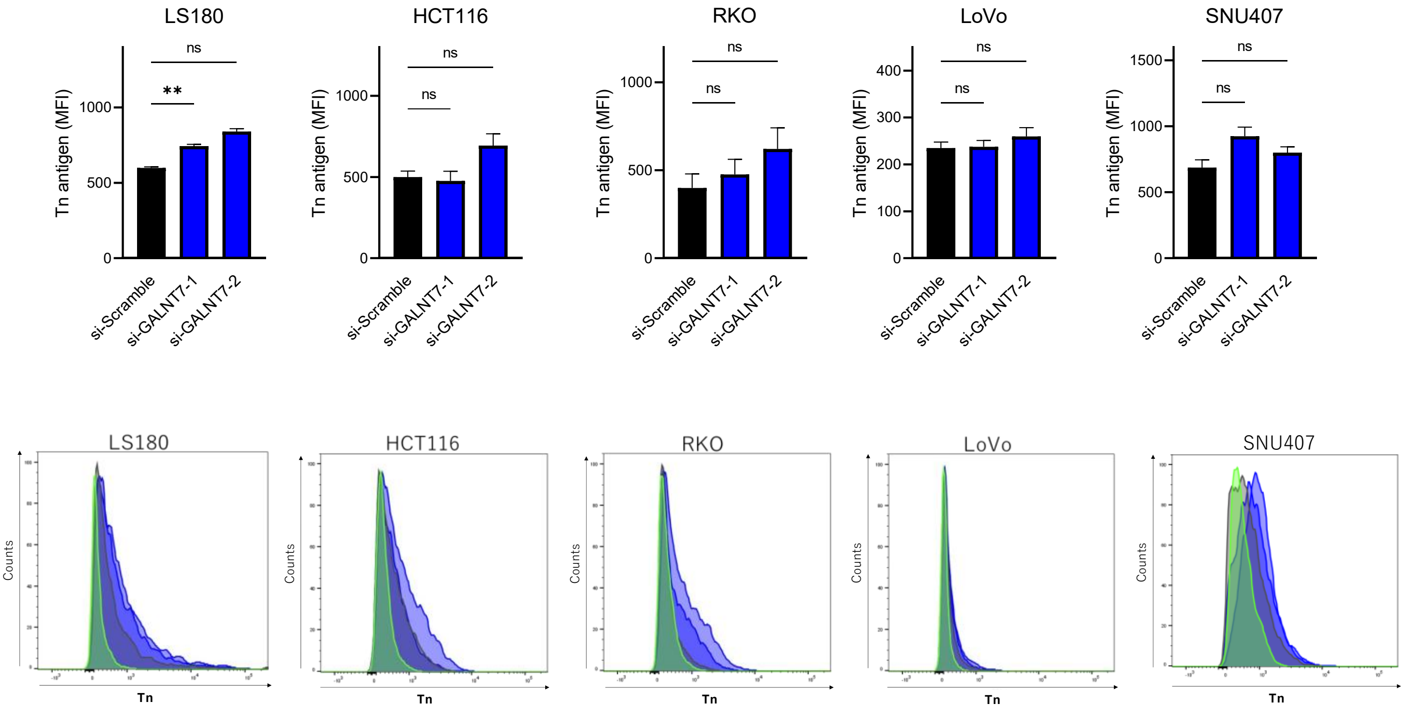

Supplementary Figure S16. Tn antigen expression analyzed by flow cytometry in CRC cell lines transfected with siRNAs targeting scramble negative control or GALNT7.
